# Bee species perform distinct foraging behaviors that are best described by different movement models

**DOI:** 10.1038/s41598-022-26858-9

**Published:** 2023-01-02

**Authors:** Johanne Brunet, Qi Jiang, Yang Zhao, Margaret W. Thairu, Murray K. Clayton

**Affiliations:** 1 Vegetable Crops Research Unit, United States Department of Agriculture-Agricultural Research Service, Madison, WI 53706 USA; 2 Department of Statistics, University of Wisconsin, Madison, WI 53706 USA; 3 Department of Entomology, University of Wisconsin, Madison, WI 53706 USA; 4 Present Address: Goldman Sachs, 200 West Street, New York, NY 10282 USA; 5 Present Address: Gilead Sciences, 333 Lakeside Dr, Foster City, CA 94402 USA; 6 Present Address: Department of Bacteriology, University of Wisconsin, Madison, WI USA

**Keywords:** Behavioural ecology, Animal behaviour, Entomology

## Abstract

In insect-pollinated plants, the foraging behavior of pollinators affects their pattern of movement. If distinct bee species vary in their foraging behaviors, different models may best describe their movement. In this study, we quantified and compared the fine scale movement of three bee species foraging on patches of *Medicago sativa.* Bee movement was described using distances and directions traveled between consecutive racemes. Bumble bees and honey bees traveled shorter distances after visiting many flowers on a raceme, while the distance traveled by leafcutting bees was independent of flower number. Transition matrices and vectors were calculated for bumble bees and honey bees to reflect their directionality of movement within foraging bouts; leafcutting bees were as likely to move in any direction. Bee species varied in their foraging behaviors, and for each bee species, we tested four movement models that differed in how distances and directions were selected, and identified the model that best explained the movement data. The fine-scale, within-patch movement of bees could not always be explained by a random movement model, and a general model of movement could not be applied to all bee species.

## Introduction

The vast majority of flowering plants are pollinated by insects. Pollinating insects move between flowers, picking up pollen from one flower and depositing it on the next flower visited. Different aspects of pollinator behavior, such as the distances and directions traveled between racemes and between plants^1–3^, and the number of flowers visited within a foraging bout or residence^4^, will influence how far a pollinator moves, together with the pollen it carries. A foraging bout represents one pollinator visit in a patch, and a patch is a group of plants growing in the same area and spatially separated from other groups of plants. Pollinators exhibiting directionality of movement, where directions of successive flight segments are correlated within foraging bouts, tend to move farther net distances relative to pollinators that move randomly among flowers^1,5^. A net distance describes the distance between where a pollinator starts and ends foraging in a patch; it is the direct line between the first and last flowers or inflorescences visited in a foraging bout. The net distances traveled by pollinators will influence the distances traveled by the pollen they carry and the resulting seeds. It is therefore important to understand pollinator movement because it influences how pollinators affect pollen dispersal and gene flow.

Modeling animal movement has been an important goal for animal ecologists, and studies have recognized the importance of linking behavior to models of animal movement^6,7^. For many animal species, models of movement are built from estimated distances and directions traveled based on telemetry data, and different statistical methods exist to analyze individual animal tracking data, depending on the type of movement data available^8,9^. These modeling approaches tend to examine the movement of larger animals over the landscape^9^. For bees, harmonic radars have been successfully used to accumulate location data every three seconds, and with a position precision within ± 3 m, at least on flat terrains without obstacles^10–12^. These data have provided useful information on various aspects of bee behavior, including their foraging range^11^, the ontogeny of bumble bee flight trajectories^12,13^, and dispersal patterns of bumble bee queens after hibernation^14^. The location data obtained with harmonic radars provide information on larger-scale movements of bees.

To fully understand bee movement, however, one must consider not only the behavioral rules used by bees at a larger scale, but also the rules used at the smaller, local scale^7^. This fine-scale movement describes bees moving from flower to flower, inflorescence to inflorescence, and plant to plant, movement patterns that occur at a scale typically less than three meters^2,15^. Fine-scale movement best delineates bees moving within a patch or an agricultural field, considered continuous landscapes. Previous studies at this smaller scale have examined how reward quantity and quality^15–17^, and floral and plant traits, including floral display size and flower color^18,19^ affect plant, inflorescence, and flower choices, together with the role of learning in these selection processes^20^.

Various studies have examined the development of traplines (multi-destination routes) by bees, both empirically and via models that examined factors affecting the process^21–24^. Methods of resource partitioning by traplining bees has also been recently modeled^25^. While traplining aimed at describing bees moving among flowers, the low number of artificial flowers or feeding stations used to empirically examine the process (< 10), and the associated modeling, may best describe movement among patches^23,26^, and thus movement at a larger scale^23^.

Here, we examine the fine scale movement of three bee species that pollinate *Medicago sativa* flowers and, for each bee species, identify the model that best describes their movement. The three bee species are the honey bee, *Apis mellifera* L., the common eastern bumble bee, *Bombus impatiens Cresson*, and the alfalfa leafcutting bee, *Megachile rotundata* F. We examine fine-scale movement of bees on plants that can each bear 100–1000 flowers^19^, and do not control for any plant traits such as floral display size. To describe within patch movement, we measured distances and directions traveled between consecutive racemes, and number of flowers visited per raceme, and for each bee species, we tested four models of bee movement. Each model differed in the method used to select distances and directions traveled between consecutive racemes (inflorescences) (Table 1). The best model for each bee species was identified using a randomization approach. Results indicated differences among bee species in their foraging behaviors, and distinct models best explaining bee movement for the different species.

**Table 1:** The four models of bee movement.

Model	Distance	Direction
Model I: Random distance-random direction	Distance obtained from the distribution of distances traveled between consecutive racemes for a bee species	Direction obtained from the distribution of directions traveled between consecutive racemes for a bee species
Model II: Random distance-modeled direction	Distance obtained from the distribution of distances traveled between consecutive racemes for a bee species	Direction determined using the matrix of transition probabilities or the transition vector for a bee species
Model III: Modeled distance-random direction	Distance obtained from the best statistical model for distance for a bee species	Direction obtained from the distribution of directions traveled between consecutive racemes for a bee species
Model IV: Modeled distance-modeled direction	Distance obtained from the best statistical model for distance for a bee species	Direction determined using the matrix of transition probabilities or the transition vector for a bee species

## Results

### Model for the distance traveled between consecutive racemes

We observed 308 foraging bouts and 2183 entries over two years (Table 2; Supplementary Table S1 online). In the first year of observations, the best models for the distance traveled between consecutive racemes did not include bee species (Supplementary Table S2 online), while bee species was statistically significant the second year (p = 0.0018) (Supplementary Table S3 online). After examining the results for each year, over all bee species (Supplementary Tables S2 and S3 online), and by bee species (Supplementary Tables S4 and S5 online), we selected the model log_e_ distance = log_e_ flower number as a good fit to the data. Flower number was a fixed effect and patch number and foraging bout were random effects in the model. Foraging bout was used as a random effect in the model, but not bee, because individual bees were not marked in the experiment. Generally, we found similar model fits whether a random foraging bout (run) effect or a repeated measures (AR(1) structure) was used in the model. We opted for a random foraging bout effect because it more easily facilitated the development of the simulation model.

**Table 2:** Sample sizes per bee species per year for foraging bouts and clips.

	Bumble bee	Honey bee	Leacutting bee	Total
**Year 1**				
Foraging bouts	79	41	37	157
Clips	751	205	86	1042
**Year 2**				
Foraging bouts	70	28	53	151
Clips	658	315	168	1141

A foraging bout includes all the racemes visited in one visit to the patch by a bee. A clip represents two successively visited racemes.

The model log_e_ distance = 3.24 −0.282 log_e_ flower number (p < 0.0001) best fitted the first-year data. Bees traveled shorter distances to the next raceme when more flowers were visited on a raceme. The random effect of foraging bout was statistically significant (estimate = 0.28, p < 0.0001) indicating variation among foraging bouts in the relationship between log_e_ flower and log_e_ distance. Adding bee species as a fixed effect and patch number as a random effect did not improve the model and the same model applied to all three bee species the first year.

The second year, bee species was statistically significant, and we found a model for each bee species. The model for bumble bees was log_e_ distance = 3.04 − 0.28 log_e_ flower number, with a statistically significant random foraging bout effect (Table 3). The negative relationship between log_e_ distance and log_e_ flower number was maintained in the honey bee model but the random foraging bout effect was not statistically significant (Table 3). For leafcutting bees, only the intercept was statistically significant in the model (Table 3). We also considered combining years and determined that a better model fit and higher precision was obtained by examining each year separately.

**Table 3:** Distance model for each bee species the second year.

	Estimate	SE	df	P
**Bumble bee**				
Intercept	3.04	0.07	68	< 0.001
Log_e_ flower number	− 0.28	0.09	583	0.003
Foraging bout	0.12	0.06		0.02
**Honey bee**				
Intercept	2.68	0.11	27	< 0.0001
Log_e_ flower number	− 0.29	0.13	284	0.02
Foraging bout	0.07	0.05		0.10
**Leafcutting bee**				
Intercept	3.04	0.10	52	< 0.0001
Log_e_ flower number	− 0.02	0.17	114	0.90
Foraging bout	0.00			

The variable SE represents the standard error of the estimate, df is the degree of freedom, and P, the probability. Foraging bout is a random variable and flower number a fixed effect in the model.

### Transition matrix or vector for directions traveled between consecutive racemes

The transition probability matrices calculated from field data are presented in Table 4 for bumble bees and honey bees. The transition vectors for the three bee species are presented in Table 5. For bumble bees and honey bees, the highest frequency was associated with remaining in the same direction (0° angle), which supports the directionality of movement within foraging bouts for these two bee species.

**Table 4:** Transition matrix for (a) bumble bee and (b) honey bee.

	E	NE	N	NW	W	SW	S	SE
**Bumble bee**								
E	0.229	0.096	0.139	0.054	0.084	0.066	0.181	0.151
NE	0.143	0.219	0.210	0.067	0.105	0.124	0.067	0.067
N	0.132	0.153	0.228	0.085	0.169	0.079	0.090	0.063
NW	0.071	0.091	0.182	0.192	0.212	0.081	0.141	0.030
W	0.082	0.048	0.155	0.077	0.290	0.116	0.169	0.063
SW	0.095	0.036	0.095	0.102	0.190	0.190	0.234	0.058
S	0.105	0.046	0.084	0.059	0.180	0.105	0.347	0.075
SE	0.165	0.037	0.110	0.046	0.165	0.092	0.211	0.174
**Honey bee**								
E	0.122	0.098	0.171	0.073	0.195	0.098	0.171	0.073
NE	0.175	0.150	0.100	0.150	0.100	0.125	0.200	0.000
N	0.061	0.121	0.227	0.121	0.212	0.045	0.136	0.076
NW	0.085	0.085	0.237	0.085	0.203	0.136	0.136	0.034
W	0.035	0.058	0.151	0.198	0.302	0.105	0.081	0.070
SW	0.143	0.061	0.061	0.102	0.204	0.204	0.143	0.082
S	0.068	0.054	0.189	0.122	0.122	0.095	0.257	0.095
SE	0.152	0.121	0.182	0.030	0.061	0.030	0.273	0.152

Each cell in the matrix represents the probability that a bee moves from one direction to the next. For example, bumble bees have a 0.229 probability of moving from East to East and a 0.096 probability of moving from East to North East.

**Table 5:** The transition vector for each bee species.

Bee species/rotation	0°	45°	90°	135°	180°	225°	270°	315°
Bumble bee	0.249	0.133	0.114	0.078	0.075	0.073	0.136	0.141
Honey bee	0.203	0.125	0.123	0.092	0.100	0.092	0.098	0.167
Leafcutting bee	0.125	0.125	0.125	0.125	0.125	0.125	0.125	0.125

The rotation indicates a 0°, 45°, 90° or larger angle rotation to the left from one movement between racemes to the next. For 0° rotation, the bee kept moving in the same direction while for a 180° rotation it reversed direction. A probability of 0.125 was used for each angle rotation for leafcutting bee.

### Model that best described bee movement for each bee species

For bumble bees, the “Modeled Distance-Modeled Direction” model was not rejected (higher probability values), whether each year was considered separately, or data from both years were combined. In contrast, the “Random Distance-Random Direction” model was repeatedly rejected (Table 6). The “Random Distance-Modeled Direction” model was generally rejected, and the “Modeled Distance-Random Direction” model was not rejected the first year, but it was rejected the second year and over both years combined, at least at the p = 0.10 level (Table 6). Based on these results, we conclude that the best model to describe bumble bee movement should include distance as a linear function of the number of flowers visited per raceme (Modeled Distance), and direction based on the transition vector (Modeled Direction).

**Table 6:** Testing the four models of bee movement for each bee species each year and for both years combined (see Table 1 and text for details).

Model	Seed	Year 1			Year 2			Combined years		
		Bumble bee	Honey bee	Leafcutting bee	Bumble bee	Honey bee	Leafcutting bee	Bumble bee	Honey bee	Leafcutting bee
**RDistance RDirection**	1	0.02	0.417	0.254	0.030	0.464	0.250	0.004	0.374	0.162
	2	0.02	0.436	0.258	0.022	0.468	0.226	0.012	0.354	0.160
	3	0.018	0.419	0.258	0.020	0.454	0.233	0.002	0.356	0.162
	4	0.014	0.432	0.248	0.030	0.473	0.223	0.004	0.350	0.142
	5	0.018	0.423	0.252	0.031	0.465	0.237	0.004	0.364	0.146
**RDistance MDirection**	1	0.088	0.438	0.218	0.051	0.448	0.228	0.026	0.414	0.160
	2	0.086	0.438	0.226	0.058	0.475	0.216	0.038	0.364	0.142
	3	0.086	0.442	0.222	0.064	0.460	0.214	0.028	0.388	0.148
	4	0.084	0.442	0.226	0.063	0.446	0.214	0.036	0.386	0.142
	5	0.095	0.434	0.239	0.055	0.460	0.220	0.032	0.354	0.140
**MDistance RDirection**	1	0.198	0.373	0.445	0.056	0.362	0.473	0.058	0.484	0.480
	2	0.203	0.366	0.432	0.063	0.376	0.463	0.07	0.482	0.442
	3	0.202	0.364	0.436	0.048	0.358	0.489	0.052	0.48	0.488
	4	0.202	0.343	0.419	0.042	0.378	0.462	0.074	0.482	0.434
	5	0.225	0.372	0.447	0.048	0.372	0.492	0.056	0.49	0.454
**MDistance MDirection**	1	0.386	0.350	0.426	0.106	0.286	0.472	0.180	0.480	0.464
	2	0.395	0.352	0.412	0.106	0.306	0.478	0.176	0.500	0.450
	3	0.372	0.355	0.408	0.096	0.286	0.495	0.154	0.480	0.468
	4	0.364	0.342	0.394	0.092	0.310	0.478	0.164	0.476	0.432
	5	0.390	0.368	0.428	0.098	0.309	0.490	0.154	0.490	0.462

The one tailed probability values (p) are presented for the randomization tests. A low probability indicates that the model is not a good fit to the data. The different seeds represent different starting points and test the robustness of the different models.

For honey bees or leafcutting bees, none of the four models were rejected, and this was true for each year separately, or for both years combined (Table 6). These results indicate that there was no evidence that the models differed in their ability to describe the movement of honey bees and leafcutting bees. In other words, sampling distances and directions from the empirical distributions provided similar results to using the best model to explain distance traveled and using the transition vector to generate directions. Because we had fewer empirical observations for honey bees and leafcutting bees relative to bumble bees each year, we reran the four models for bumble bees, using sample sizes comparable to honey bees, and could no longer reject any of the four models (Suppl. Table S6). A subsample of the frequency distributions of mean net distances traveled for the “Random Distance-Random Direction” model, based on 2000 simulations, for each bee species, each year, is presented in Supplementary Figure S1 online.

## Discussion

The approach used in this study differs from previously published models such as BEEHAVE^27^, and BEESCOUT^28^. The aim of BEEHAVE is to assert the impact of distinct factors, including pesticides, diseases, changes in landscape structure, and foraging on honey bee colony health and growth, while BEESCOUT aims more at determining the probabilities of bees detecting food sources over the landscape based on the configuration of the landscape and on the bee search behavior. In this study, we examined the fine scale movement of bees foraging in a patch, and identified the best model of bee movement for each of three bee species. In this respect, the models introduced here share some similarities with Rands^7^, but in the current study, data on observed bee foraging behavior of distinct bee species are used to select best models of bee movement. The general approach follows Levey et al.^29,30^ who use perching time, move length, and move direction to describe small-scale bird movement. In the current study, distances and directions travelled between consecutive racemes by bees are used to parameterize the models of bee movement, without the need for data error correction^31^. Results indicate differences in foraging behaviors among bee species, and differences in models of bee movement.

For bumble bees, we obtained a large enough sample size to successfully discriminate among the four distinct movement models. The “Modeled Distance-Modeled Direction” model best explained bumble bee movement. Bumble bees show directionality of movement within foraging bouts^1^ and the number of flowers visited on a raceme affects the distance traveled to the next raceme (results therein). These foraging behaviors improved predictions of the movement of bumble bees over a continuous landscape, and this supports the importance of linking animal behavior to model of animal movement^6,31^.

For leafcutting bees, none of the four models could be rejected. In retrospect, however, all four models for leafcutting bees make similar predictions and reflect a pattern of random movement. For example, for leafcutting bees, only the intercept was significant in a model of the distance traveled to the next raceme (“Modeled Distance”). This means that distances traveled can be described by a normal distribution with an estimated mean and variance, which is quite similar to randomly selecting distances from a distribution (“Random Distance”). In addition, the matrix of transition probabilities (“Modeled Direction”) had an equal probability of moving in any of the directions, because leafcutting bees did not exhibit directionality in their pattern of movement within foraging bouts^1^. This is not very different from randomly selecting each direction from the distribution of directions (“Random Direction”). In other words, all four models made fairly similar predictions with respect to bee movement, which all reflected “Random Distance” and “Random Direction”. The four models could therefore not be distinguished and the data suggest a model of “Random Distance-Random Direction” as the most likely model to describe leafcutting bee movement.

We could not discriminate among the different models for honey bees, likely due to the lower sample sizes providing less statistical power. This conclusion is supported by the fact that we could not discriminate among the different models when we reduced the sample size of bumble bees to the sample size observed for honey bees. Moreover, we still could not discriminate among models when combining data from both years for honey bees. Combining years for honey bees provided a similar number of foraging bouts, but still fewer clips (520 relative to 751 or 658) compared to one year for bumble bees. Like bumble bees, honey bees exhibit directionality of movement within foraging bouts^1^ and they travel shorter distances to the next raceme after visiting more flowers on a raceme (results herein). Based on the results obtained for bumble bees, we propose the “Modeled Distance-Modeled Direction” model as the most likely model to describe honey bee movement. This assumes the model would best explain the data were sample sizes to be larger.

When we examined solely the “Modeled Distance” portion of the model, a bumble bee or a honey bee traveled a shorter distance to the next raceme when more flowers were visited on a raceme and the distance increased when fewer flowers were visited. This information suggests bumble bees and honey bees can assess resource availability and this information influences their movement. Many factors can affect floral resource availability, including recent visits to flowers by bees^32^, which may be detectable via scent marks^33,34^. Bees may visit more flowers on racemes that provide good resources, and travel shorter distances after visiting more flowers on a raceme, potentially expecting to find other profitable neighboring racemes in the vicinity, either on the same or on a different plant. Bumble bees can identify flowers that offer pollen^17^ and both bumble bees and honey bees prefer inflorescences with more flowers^35,36^. Moreover, both bumble bees and honey bees prefer inflorescences with more pollen-producing flowers when foraging for pollen, and inflorescences with more nectar-producing flowers when they forage for nectar^17,37,38^. Using artificial flowers presenting nectar as a reward, Waddington^39^ reports bumble bees traveling short distances after visiting rewarding flowers, but the distance did not vary with the number of rewarding flowers visited. However, the distance traveled by the bee increased with the number of non-rewarding flowers visited. Lihoreau et al.^22^ found bumble bees increase the distance traveled to visit high-reward sites but only for small departure (18%) from the shortest possible distance. Interestingly, for leafcutting bees, previously visited resources did not guide their movement to the next resource. Results of this study highlight differences in how bee species use information about previously visited resources to guide their pattern of movement. Future research should determine whether social bees, relative to solitary bees, are more likely to use information about previously visited resources in determining their next move, and why such differences may exist between groups of bees.

The models developed herein illustrate movement over continuous landscapes. Studying seabirds looking for prey, Miramontes et al.^40^ showed how the landing pattern did not resemble the search pattern, and concluded that the pattern of movement depended little on the forager behavior, but more on the spatial distribution of resources. In the current study, we compare three bee species under similar conditions (resource distribution) and find differences in the models that best explained their landing patterns. Because these models reflect different bee behaviors, such as resource information collected by the individual, and persistence of movement directionality, we conclude that bee foraging behavior affects their movement patterns. Bees have complex foraging behaviors, and flower selection does not depend solely on the spatial distribution of resources. Bees use visual and olfactory cues to select which plants to visit^19^, and learn to associate floral traits with rewards, a process called associative learning^41–43^. Resources get depleted following bee visits and the reward landscape is constantly changing. We are not claiming that resource distribution does not affect the pattern of bee movement, of course it will because bees visit plants to gather resources and do not land on the ground while foraging. However, our results indicate that resource distribution is not sufficient to explain bee movement, differences in bee foraging behavior among bee species must be considered. The interaction of bee foraging behavior with resource distribution will determine bee movement patterns.

The approach developed herein could be extended to discontinuous landscapes. Bees follow decision rules not only to select plants and inflorescences within patches but also to decide which patch to move to next^18,26^. The bee movement model over discontinuous landscapes could include two modes of movement, with bees switching between behavioral modes as they forage over the landscape^6,8^. The first mode represents bee movement within patches, and the second mode addresses bees selecting the next patch to move to. Furthermore, a third mode could be added to represent bees switching between plant species, either within or between patches. When incorporating these modes, it is important to consider that the rules followed by bees within a mode may vary among bee species. For example, bee species may follow different rules when selecting the next patch to move to. In addition, for the mode switching between plant species, a previous study examining bumble bees and honey bees foraging over the landscape detected pollen from a single plant family in 90% of the foraging trips made by honey bee individuals, but only in over 60% of the foraging trips made by bumble bees^44^. A foraging trip, the time elapsed between a bee leaving and returning to the hive, measured using Radio Frequency Identification (RFID), lasted approximately 50 min on average for both bee species^45^. While pollen was not identified at the plant species level in the study, results suggest potential differences in plant species fidelity between the two bee species. A clear message from the current study is the importance of considering differences in foraging behavior among bee species when developing models of bee movement, and that a general movement model cannot be applied to all bee species.

## Conclusions

For some bee species, bee movement cannot be explained by a simple random movement model, but resource information collected by the individual, and persistence of movement directionality must be considered. Bee species differ in foraging behaviors that affect movement, and different models best describe movement for distinct bee species. A general movement model should not be applied to all bee species.

## Methods

### Plant species and pollinators

*Medicago sativa* L. (Fabaceae), also called alfalfa or lucerne, is a perennial legume with flowers arranged in a cluster or raceme. It is a self-compatible plant with fairly high outcrossing rate (5.3–30%)^46^, and it requires insect visits for seed production^47^. No plant material was collected for this study. Honey bees, *Apis mellifera*, and alfalfa leafcutting bees, *Megachile rotundata*, are used as managed pollinators in alfalfa seed-production fields in the USA while bumble bees are commonly used in alfalfa breeding^47^.

### Experimental design and pollinator observations

Five 11 m × 11 m patches of *M. sativa* plants were set up in an east–west linear arrangement at the West Madison Agricultural Research Station in Madison, Wisconsin, USA. Within each patch, we transplanted 169 young plants grown from seeds in the greenhouse, each placed 90 cm apart. These plants grew and, at flowering, a plant had an average of 30.65 ± 16.4 stems per plant, with 4.93 ± 3.41 racemes per stem, and 7.53 ± 2.44 open flowers per raceme.

A honey bee hive was placed approximately 100 m from the patches and a bumble bee hive was set up at the center of the southern edge of the patches. For leafcutting bees, a 60 × 30 × 7.6 cm bee board was set up in each of two boxes placed 1/3 and 2/3 along the southern edge of the patches and a half gallon of bees was released at periodic intervals throughout the alfalfa flowering season.

**Figure 1:**
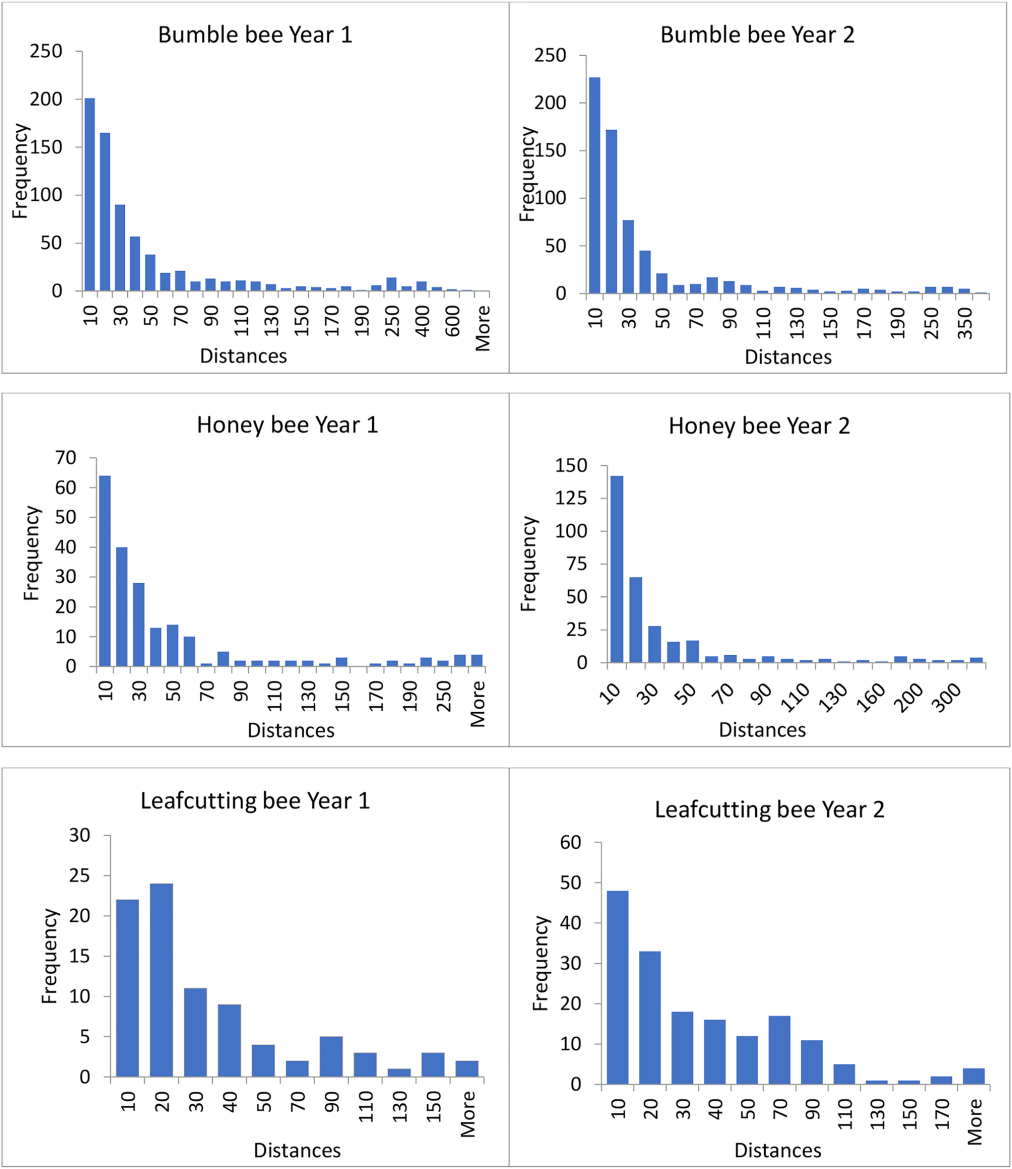
Frequency distributions for distances traveled between consecutive racemes (cm) for each bee species each year.

Over two consecutive summers, observers followed bees foraging in the alfalfa patches, marked each raceme visited in succession within a foraging bout with a numbered clip, and recorded the number of flowers visited per raceme. After a bee had left a patch, observers went back to the marked racemes and measured the distance and direction traveled between consecutive racemes. Directions were recorded as one of the cardinal directions: North (N), South (S), East (E) or West (W), or inter-cardinal directions: Northeast (NE), Southeast (SE), Northwest (NW) and Southwest (SW). The frequency distributions of distances and directions traveled between two successive racemes are presented for each bee species each year in Figs. 1 (distances) and 2 (directions). The low pollinator abundance permitted observers to follow individual bees foraging in a patch. Little interference among bee species was observed in the patches.

**Figure 2:**
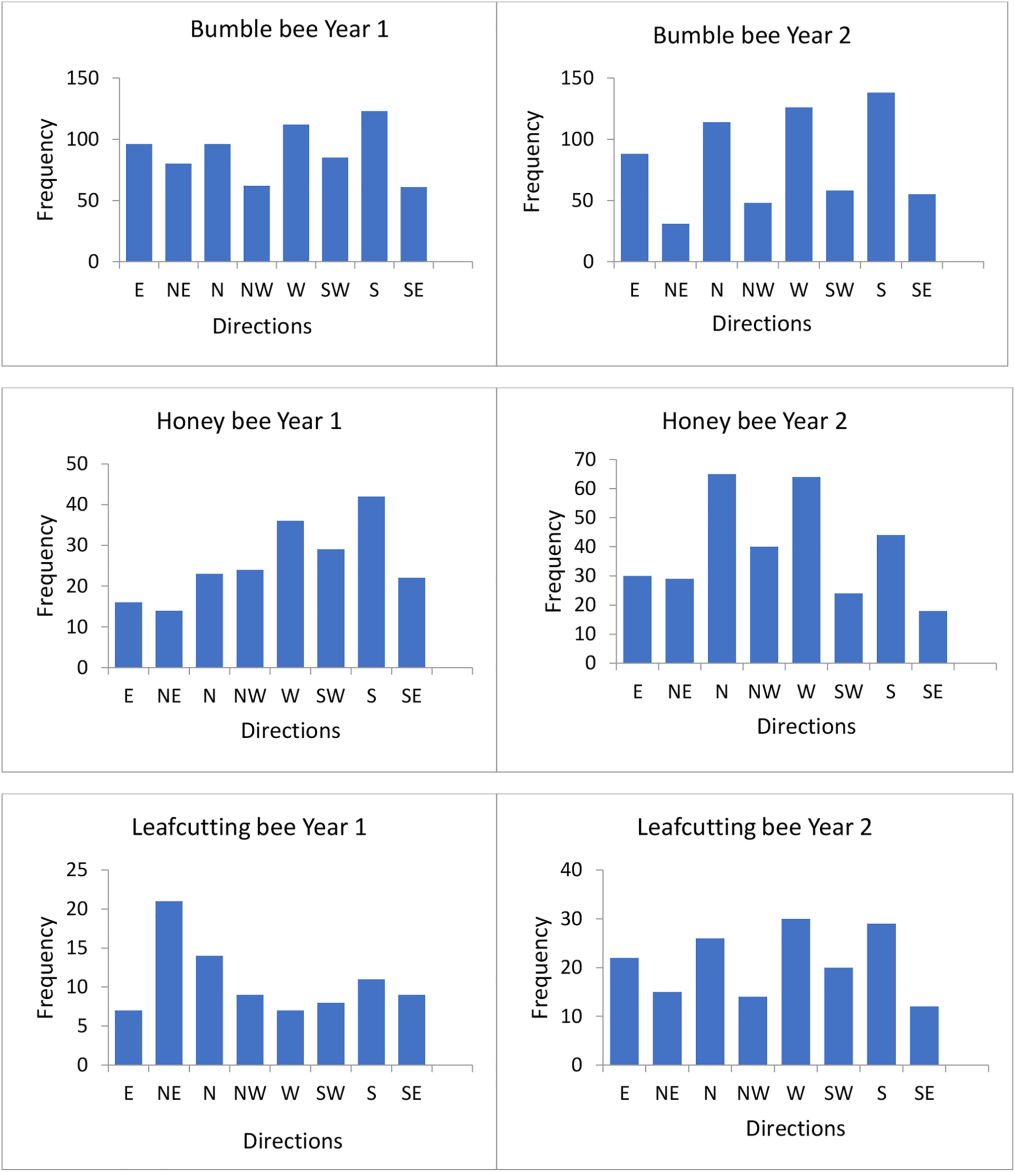
Frequency distributions of directions traveled between consecutive racemes for each bee species each year.

### Model for the distance traveled between consecutive racemes

We first determined whether a statistical model best described the distance traveled between consecutive racemes (Modeled Distance), and examined whether the model differed among bee species. We used mixed effect linear models (proc Mixed in SAS 9.3)^48^ to identify the model that best described the distance traveled by pollinators between consecutive racemes. The model included log_e_ distance as a linear function of log_e_ flower number and bee species as fixed effects. The distance traveled between consecutive racemes and the number of flowers visited per raceme were log transformed prior to analyses in order to improve the models’ residuals. In addition, we included patch and foraging bout as random effects in the model. A foraging bout includes the racemes visited in succession from the time a bee is spotted in a patch to the time it leaves that patch. We used foraging bout instead of individual bee as the random effect because bees were not individually marked in this study. Moreover, to take into consideration the potential correlation between successive observations within a foraging bout, we added clip to the model. Clip 1 represents the first and second racemes visited in the foraging bout; clip 2, the second and third, and so on. Clip was added to the model either as a random effect or as a repeated measure with an AR(1) structure. The combination of random clip and random foraging bout creates a model that is sometimes called the “compound symmetry” model. The AR(1) structure represents correlations that decline exponentially as the gap between measurements increases such that measurements closer together in time are more strongly correlated than measurements further apart. Because we expected bees to visit flowers at close proximity when resources are abundant, we chose this correlation structure as a good potential descriptor of the way distances might be correlated within foraging bouts. We started with a full model which included log_e_ flower number, bee species, patch, foraging bout, and clip either as a random effect or as a repeated measure with an AR(1) structure. We then removed variables and compared models by inspecting AIC values and the p values for each term in the model. We considered both low AIC and statistically significant (p < 0.05) terms for model selection. We examined each year separately, and within each year, determined whether bee species affected the model and, thus, whether we needed a separate model for each bee species.

### Transition matrix or vector for directions traveled between consecutive racemes

We computed matrices of transition probabilities based on field-collected data for bumble bees and honey bees because these two bee species exhibited directionality of movement within foraging bouts^1^. These matrices represent the probability that a bee moved from one direction to the next when visiting two consecutive pairs of racemes (Modeled Direction). We recorded eight potential directions in the field which lead to a transition matrix with 64 cells. For a given bee species, each year, the transition probability for a cell in the matrix was calculated by dividing the number of transitions (counts) within a cell in a particular row by the total number of transitions for that row such that each row's total frequency was equal to 1.0. Because leafcutting bees do not exhibit directionality of movement within foraging bouts^1^, given the eight possible directions, we assigned an equal probability of 0.125 of moving from one direction to the next for all cells of the matrix.

Because a matrix of transition probabilities contained 64 cells, and therefore 64 probabilities to estimate, we alternatively calculated transition vectors which reduced the number of transitions to estimate. The eight cells in the transition vector represented, respectively, the probability that a bee remained in the same direction (0), turned left 45°, 90°, 135°, 180°, 225°, 270° or 315° angles. For example, if a bee moved south, north, northeast and then northwest during its foraging bout, this represented, respectively, a 180° (south to north), 315° (north to northeast), and 90° (northeast to northwest) left angles. To obtain the probability for each of the eight cells of the transition vector, we summed the number of times a bee moved a given angle and divided that number for each angle by the total number of transitions for that bee species. This frequency table was used as an approximation of the transition vector for bumble bees and honey bees. For leafcutting bees, we assigned a probability of 0.125 to each cell of the vector. Because, for each bee species, the transition probability matrices or the transition vectors were very similar between years, we combined data from both years to calculate them, which increased sample sizes.

### Modeling pollinator movement

We examined models simulating a path along which a bee traveled for each foraging bout (Supplementary Fig. S2). A foraging bout included all racemes visited by a bee during one visit to the patch. Bee movement was modeled by the distance traveled and the direction of movement between consecutive racemes. Starting from the origin (0, 0), the first move was simulated by randomly selecting a direction amongst the eight possible directions, N, NE, E, SE, S, SW, W, NW, which corresponds to a bee showing no overall preference for a direction. Following the first move, a distance and a direction traveled between consecutive racemes were chosen each time a bee moved between consecutive racemes.

Four distinct models of bee movement were tested for each bee species. These models are referred to as the Random Distance-Random Direction model; the Random Distance-Modeled Direction model; the Modeled Distance-Random Direction model; and the Modeled Distance-Modeled Direction model (Table 1). For the Random Distance or Random Direction part of a model, the distances or directions traveled between consecutive racemes were selected randomly from the respective distribution of empirical distances (Fig. 1) or directions (Fig. 2) traveled for that bee species that year (Table 1). For Modeled Distance, distances were predicted as a function of the number of flowers visited in the previous raceme (statistical model for the distance traveled between consecutive racemes) plus a prediction error. This error was generated from a normal distribution whose spread was based on the residuals’ standard deviation. A separate equation was used for each bee species. For Modeled Direction, we used the transition matrix or transition vector for the bee species. When using the transition vector, directions were simulated using the frequency distribution of potential transitions. The length of a foraging bout was selected randomly from the empirical distribution of foraging bout lengths obtained for a bee species in a given year without replacement.

### Selecting the best movement model for each bee species

To select the best movement model, we contrasted the empirical mean or median net distance traveled by a bee species in a given year, against the distribution of mean or median net distances generated for each of the four models for that bee species that year. The net distance is the straight-line distance between the first- and the last- visited raceme of the foraging bout and this measure relates well to pollen dispersal and gene flow. While many movement models have used mean-square displacements^49,50^, which describes an average distance traveled per unit of time, we used the average (or median) net distance traveled. No one- or two-dimensional measure necessarily captures all features of a movement model and the mean and/or median permitted us to distinguish among some of the models. We tabulated the mean and median empirical net distances traveled, over all observed foraging bouts, for a bee species each year. This approach generated six observed means and six observed medians, one for each of three bee species and two years.

For simulated net distances, we followed a similar approach but here distances and directions were generated differently depending on which of the four models was being tested (Table 1). For each bee species each year, and for each of the four models, we simulated 2000 mean and median net distances traveled, and thus, 24 sets of 2000 mean and median net distances traveled (4 models × 3 bee species × 2 years). In addition, we obtained 500 mean and median net distances traveled for combined years for each bee species.

A randomization approach was used to identify the model that best fitted the observed data. The p value was determined by counting the percentage of time, out of 2000, the simulated mean or median net distance was greater or smaller than the observed mean or median distance traveled for the bee species that year. With three bee species, 2 years, and four models, we performed 24 randomization tests. When testing the observed mean to the simulated distribution of means, a low probability value indicates rejection of the model. To determine the robustness of our findings, we repeated each randomization test using five different starting points, i.e. random number seeds. This was done using the set.seed random number generator function in R 3.5.1.

Our ability to reject or accept a given model was similar whether we used the median or the mean distance traveled between consecutive racemes. Because the mean may better reflect the skewness of the distributions of distances traveled, we present the results obtained using the mean net distance traveled. Moreover, few differences existed between the results of the simulation models based on transition matrices or transition vectors, and fewer parameters needed to be estimated for transition vectors, therefore parameter estimates of the transition vectors were more stable for a given sample size, and we present the results of the simulation models obtained using transition vectors.

## Supplementary Information

Supplementary Information 1.

Supplementary Information 2.

## Data Availability

Data are available at 10.5061/dryad.m7f92c3 and in online Supplementary Information Table S1.
