# Supplementary material for: Bee species perform distinct foraging behaviors that are best described by different movement models
Source: Sci Rep. 2023 Jan 2;13:71. doi: 10.1038/s41598-022-26858-9 (PMC9807645; doi:10.1038/s41598-022-26858-9)
Supplement: Supplementary file 1 — Supplementary Information 1. [file 41598_2022_26858_MOESM1_ESM.pdf]

Bee species perform distinct foraging behaviors that are best described by different movement models

Johanne Brunet[1], Qi Jiang[2$], Yang Zhao[2+], Margaret W. Thairu[3#] and Murray K. Clayton[2]

[1] Vegetable Crops Research Unit, United States Department of Agriculture-Agricultural Research Service, Madison, WI 53706, United States

[2] Department of Statistics, University of Wisconsin, Madison, WI 53706, USA

[3] Department of Entomology, University of Wisconsin, Madison, WI 53706, USA

$ Current address: Goldman Sachs, 200 West Street, New York, NY 10282

[+] Current address: Gilead Sciences, 333 Lakeside Dr, Foster City, CA 94402

[#] Current address: Department of Bacteriology, University of Wisconsin, Madison, WI.

Corresponding author: Johanne Brunet Johanne.Brunet@usda.gov

Supplementary Table S1. Distance (Dist) (cm) and direction (dir) traveled by a bee and number of flowers (flwnum) visited per raceme for bumble bee (B), honey bee (HB) and leafcutting bee (LCB) for each clip (two consecutive racemes) in a foraging bout. A foraging bout includes all the racemes visited in a patch.

year	type	Clip	Dist	dir	flwnum
1	B	1	20	SW	8
1	B	2	8	SW	1
1	B	3	6.5	W	5
1	B	4	NA	NA	1
1	B	1	5	E	1
1	B	2	10	S	1
1	B	3	37	N	2
1	B	4	5	S	2
1	B	5	14	SW	2
1	B	6	5	N	4
1	B	7	8	SE	1
1	B	8	8	S	1
1	B	9	3	E	4
1	B	10	5	S	3
1	B	11	15	S	1
1	B	12	NA	NA	1
1	B	1	25	SE	3
1	B	2	53	S	1
1	B	3	5	E	2
1	B	4	2	E	3
1	B	5	2	E	1
1	B	6	23	SW	1
1	B	7	61	E	4
1	B	8	4	E	4
1	B	9	12	W	1
1	B	10	18	N	1
1	B	11	4	SW	1
1	B	12	2	S	3
1	B	13	20	NW	1
1	B	14	15	S	4
1	B	15	40	S	1
1	B	16	10	W	2
1	B	17	18	W	1
year	type	Clip	Dist	dir	flwnum
1	B	18	17	E	4

1	B	19	18	SW	1
1	B	20	48	NW	2
1	B	21	45	NW	1
1	B	22	8	W	2
1	B	23	8	S	5
1	B	24	16	S	1
1	B	25	4	SW	1
1	B	26	20	W	1
1	B	27	12	N	1
1	B	28	40	E	2
1	B	29	NA	NA	1
1	B	1	33	W	2
1	B	2	42	W	2
1	B	3	41	W	1
1	B	4	41	W	1
1	B	5	45	NW	3
1	B	6	NA	NA	NA
1	B	1	20	W	2
1	B	2	35	NW	1
1	B	3	197	W	3
1	B	4	55	W	1
1	B	5	460	W	1
1	B	6	2	W	1
1	B	7	NA	NA	1
1	B	1	292	W	1
1	B	2	NA	NA	NA
1	B	1	5	W	1
1	B	2	5	W	1
1	B	3	5	W	1
1	B	4	12	NW	1
1	B	1	31	N	2
1	B	2	NA	NA	2
1	B	1	10	S	NA
1	B	2	NA	NA	NA
1	B	1	40	SW	1
1	B	2	130	W	3
1	B	3	30	SE	1
1	B	4	53	SW	1
1	B	5	40	NE	4
1	B	6	2	NE	1
1	B	7	10	W	2
year	type	Clip	Dist	dir	flwnum
1	B	8	13	SW	1

1	B	9	10	SW	3
1	B	10	15	NW	2
1	B	11	20	W	1
1	B	12	45	NW	1
1	B	13	29	N	1
1	B	14	2	NW	1
1	B	15	45	SW	3
1	B	16	80	S	2
1	B	17	10	SE	1
1	B	18	100	SW	1
1	B	19	20	SW	2
1	B	20	62	W	1
1	B	21	58	S	1
1	B	22	25	W	1
1	B	23	27.5	S	1
1	B	24	1.5	E	3
1	B	25	42	NW	3
1	B	26	37	SW	2
1	B	27	14	S	2
1	B	28	NA	NA	NA
1	B	1	20	NE	2
1	B	2	460	W	1
1	B	3	30	SW	3
1	B	4	20	E	1
1	B	5	NA	NA	1
1	B	1	10	SW	3
1	B	2	195	NW	3
1	B	3	48	SW	1
1	B	4	100	W	2
1	B	5	7	N	5
1	B	6	4	W	3
1	B	7	NA	NA	2
1	B	1	25	W	4
1	B	2	26	E	2
1	B	3	14	SW	2
1	B	4	10	SW	1
1	B	5	60	SW	1
1	B	6	23	E	3
1	B	7	85	SW	1
1	B	8	6	N	2
1	B	9	45	SW	1
year	type	Clip	Dist	dir	flwnum
1	B	10	20	SW	2

1	B	11	20	W	1
1	B	12	83	SW	2
1	B	13	22	SW	1
1	B	14	109	S	1
1	B	15	115	SW	13
1	B	16	265	S	1
1	B	17	10	S	1
1	B	18	20	NW	1
1	B	19	6	W	1
1	B	20	200	W	1
1	B	21	NA	NA	NA
1	B	1	70	NE	2
1	B	2	24	W	3
1	B	3	20	SE	1
1	B	4	4	N	3
1	B	5	2	SW	3
1	B	6	15	E	1
1	B	7	50	SE	1
1	B	8	10	W	4
1	B	9	5	N	4
1	B	10	10	S	3
1	B	11	30	N	1
1	B	12	8	SW	8
1	B	13	15	S	1
1	B	14	25	NW	3
1	B	15	1	NE	1
1	B	16	20	SW	3
1	B	17	20	N	2
1	B	18	1	W	3
1	B	19	10	W	2
1	B	20	57	S	2
1	B	21	160	W	3
1	B	22	20	NE	1
1	B	23	20	N	6
1	B	24	15	N	3
1	B	25	11	NW	2
1	B	26	13	NW	2
1	B	27	32	N	2
1	B	28	NA	NA	4
1	B	1	38	SW	2
1	B	2	65	W	1
year	type	Clip	Dist	dir	flwnum
1	B	3	NA	NA	3

1	B	1	35	SW	4
1	B	2	NA	NA	1
1	B	1	9	N	8
1	B	2	39	E	1
1	B	3	NA	NA	1
1	B	1	97	S	1
1	B	2	90	S	1
1	B	3	70	SE	1
1	B	4	1	S	4
1	B	5	29	N	1
1	B	6	45	S	1
1	B	7	174	SE	3
1	B	8	112	W	3
1	B	9	103	W	5
1	B	10	40	SW	2
1	B	11	47	SW	1
1	B	12	NA	NA	2
1	B	1	60	SW	2
1	B	2	220	S	1
1	B	3	68	SW	1
1	B	4	32	SW	2
1	B	5	70	W	1
1	B	6	20	SW	1
1	B	7	NA	NA	1
1	B	1	70	S	2
1	B	2	21	S	1
1	B	3	33	W	1
1	B	4	10	S	1
1	B	5	155	NW	2
1	B	6	53	NW	1
1	B	7	35	NW	3
1	B	8	30	NW	1
1	B	9	328	NW	1
1	B	10	38	W	1
1	B	11	40	W	3
1	B	12	15	W	2
1	B	13	338	SW	2
1	B	14	28	S	1
1	B	15	NA	NA	1
1	B	1	63	SE	1
1	B	2	NA	NA	2
year	type	Clip	Dist	dir	flwnum
1	B	1	70	NW	6

1	B	2	41	NW	2
1	B	3	22	S	1
1	B	4	335	SW	3
1	B	5	108	SW	2
1	B	6	145	SE	1
1	B	7	33	SW	1
1	B	8	557	S	1
1	B	9	40	S	1
1	B	10	NA	NA	2
1	B	1	4	NW	1
1	B	2	73	SE	1
1	B	3	42	S	2
1	B	4	24	W	1
1	B	5	68	W	2
1	B	6	64	W	1
1	B	7	5	S	4
1	B	8	10	S	3
1	B	9	17	NW	3
1	B	10	14	N	1
1	B	11	2	S	2
1	B	12	28	SW	1
1	B	13	8	W	2
1	B	14	27	SW	1
1	B	15	116	SW	3
1	B	16	20	W	1
1	B	17	NA	NA	1
1	B	1	18	W	5
1	B	2	12	W	6
1	B	3	16	SE	4
1	B	4	4	E	4
1	B	5	6	S	1
1	B	6	23	S	1
1	B	7	10	E	2
1	B	8	25	N	1
1	B	9	NA	NA	1
1	B	1	11	E	1
1	B	2	10	NW	1
1	B	3	NA	NA	3
1	B	1	100	SE	1
1	B	2	175	SW	1
1	B	3	357	NW	1
year	type	Clip	Dist	dir	flwnum
1	B	4	245	NW	3

1	B	5	230	NW	2
1	B	6	NA	NA	3
1	B	1	13	S	2
1	B	2	NA	NA	4
1	B	1	30	N	3
1	B	2	9	E	4
1	B	3	NA	NA	1
1	B	1	13	E	1
1	B	2	8	NE	5
1	B	3	8	NE	5
1	B	4	34	NE	3
1	B	5	2	W	4
1	B	6	17	S	4
1	B	7	15	SE	2
1	B	8	NA	NA	1
1	B	1	307	N	1
1	B	2	23	S	4
1	B	3	11	W	2
1	B	4	10	W	6
1	B	5	14	SE	1
1	B	6	31	S	1
1	B	7	13	S	2
1	B	8	31	SW	1
1	B	9	64	S	3
1	B	10	20	S	1
1	B	11	NA	NA	1
1	B	1	5	E	2
1	B	2	10	N	4
1	B	3	NA	NA	6
1	B	1	27	S	1
1	B	2	24	W	3
1	B	3	NA	NA	1
1	B	1	24	E	1
1	B	2	29	W	2
1	B	3	48	SW	2
1	B	4	14	S	3
1	B	5	42	S	2
1	B	6	65	SW	2
1	B	7	55	S	1
1	B	8	26	E	1
1	B	9	NA	NA	2
1	B	1	83	NE	1
1	B	2	6	W	2

1	B	3	7	S	1
1	B	4	NA	NA	1
1	B	1	8	S	1
1	B	2	17	NE	NA
1	B	3	2	S	3
1	B	4	11	S	2
1	B	5	12	SW	9
1	B	6	11	W	3
1	B	7	7	S	2
1	B	8	26	E	1
1	B	9	15	S	1
1	B	10	7	NW	1
1	B	11	5	S	1
1	B	12	17	SW	1
1	B	13	15	N	1
1	B	14	30	N	3
1	B	15	NA	NA	2
1	B	1	276	NE	1
1	B	2	6	N	2
1	B	3	40	E	3
1	B	4	30	SE	1
1	B	5	20	SE	3
1	B	6	6	SW	2
1	B	7	270	SE	2
1	B	8	24	S	2
1	B	9	28	W	3
1	B	10	24	NE	5
1	B	11	NA	NA	1
1	B	12	NA	NA	3
1	B	13	9	NW	2
1	B	14	12	SW	2
1	B	15	45	SE	1
1	B	16	13	E	3
1	B	17	25	SE	4
1	B	18	67	SE	1
1	B	19	3	E	4
1	B	20	185	N	6
1	B	21	13	SW	6
1	B	22	11	SW	3
1	B	23	145	NW	2
1	B	24	25	N	1
1	B	25	35	SW	6
1	B	26	103	W	1

1	B	27	20	E	2
1	B	28	33	S	2
1	B	29	13	S	1
1	B	30	5	W	2
1	B	31	14	S	1
1	B	32	35	S	1
1	B	33	11	W	4
1	B	34	7	W	1
1	B	35	103	N	1
1	B	36	36	NW	2
1	B	37	7	E	2
1	B	38	NA	NA	1
1	B	1	205	E	4
1	B	2	43	SE	2
1	B	3	44	SE	1
1	B	4	24	E	2
1	B	5	119	SE	3
1	B	6	31	SE	3
1	B	7	4	N	1
1	B	8	25	N	4
1	B	9	22	SE	2
1	B	10	NA	NA	2
1	B	1	9	E	1
1	B	2	26	N	2
1	B	3	51	SW	2
1	B	4	NA	NA	1
1	B	1	13	E	1
1	B	2	10	NE	1
1	B	3	NA	NA	1
1	B	1	2	NW	2
1	B	2	41	S	3
1	B	3	26	N	1
1	B	4	3	E	4
1	B	5	65	NW	2
1	B	6	27	N	1
1	B	7	3	SW	4
1	B	8	10	NW	1
1	B	9	14	S	2
1	B	10	20	W	2
1	B	11	28	SW	3
1	B	12	17	S	1
1	B	13	12	N	4
1	B	14	3	N	1

1	B	15	9	W	3
1	B	16	15	E	3
1	B	17	3	NE	1
1	B	18	3	SW	1
1	B	19	3	NE	1
1	B	20	18	N	2
1	B	21	17	E	1
1	B	22	6	S	2
1	B	23	5	E	1
1	B	24	11	NE	3
1	B	25	3	E	1
1	B	26	10	W	3
1	B	27	NA	NA	1
1	B	1	27	W	1
1	B	2	2	N	1
1	B	3	27	NE	4
1	B	4	19	N	1
1	B	5	45	E	2
1	B	6	109	E	5
1	B	7	NA	NA	2
1	B	1	325	SE	2
1	B	2	126	S	2
1	B	3	326	SW	2
1	B	4	401	W	2
1	B	5	308	N	1
1	B	6	553	NW	2
1	B	7	175	NE	1
1	B	8	5	N	1
1	B	9	42	NE	1
1	B	10	627	E	1
1	B	11	90	NE	1
1	B	12	111	NE	3
1	B	13	235	NE	1
1	B	14	NA	NA	2
1	B	1	60	E	1
1	B	2	64	E	2
1	B	3	80	SE	1
1	B	4	20	W	6
1	B	5	11	S	1
1	B	6	9	S	2
1	B	7	18	SE	2
1	B	8	7	W	2
1	B	9	10	E	10

1	B	10	25	SW	1
1	B	11	12	NW	2
1	B	12	NA	NA	1
1	B	1	112	SW	1
1	B	2	130	SW	1
1	B	3	14	S	6
1	B	4	15	S	1
1	B	5	20	SW	3
1	B	6	11	N	1
1	B	7	15	NE	1
1	B	8	48	SW	1
1	B	9	91	NW	1
1	B	10	5	NW	1
1	B	11	2	S	9
1	B	12	4	E	3
1	B	13	5	NW	1
1	B	14	25	W	7
1	B	15	4	S	5
1	B	16	4	NE	3
1	B	17	3	NW	3
1	B	18	18	E	2
1	B	19	35	SE	2
1	B	20	10	E	4
1	B	21	8	S	4
1	B	22	149	NW	2
1	B	23	21	N	3
1	B	24	15	NE	1
1	B	25	NA	NA	1
1	B	1	40	N	1
1	B	2	13	E	3
1	B	3	30	S	1
1	B	4	5	W	3
1	B	5	4	N	3
1	B	6	NA	NA	1
1	B	1	10	N	3
1	B	2	10	N	2
1	B	3	NA	NA	1
1	B	1	25	N	1
1	B	2	20	N	1
1	B	3	20	N	3
1	B	4	95	E	1
1	B	5	200	E	1
1	B	6	17	S	1

1	B	7	NA	NA	2
1	B	1	14	SW	4
1	B	2	2	SW	3
1	B	3	NA	NA	1
1	B	1	13	NW	3
1	B	2	91	W	1
1	B	3	15	NW	1
1	B	4	80	NW	3
1	B	5	26	NE	1
1	B	6	10	NE	2
1	B	7	4	E	2
1	B	8	5	E	1
1	B	9	NA	NA	6
1	B	1	5	N	3
1	B	2	21	W	1
1	B	3	NA	NA	1
1	B	1	20	NW	1
1	B	2	55	NE	2
1	B	3	12	N	4
1	B	4	22	S	1
1	B	5	15	N	1
1	B	6	75	NE	1
1	B	7	46	E	2
1	B	8	150	NE	1
1	B	9	20	W	1
1	B	10	15	N	2
1	B	11	39	NE	1
1	B	12	33	N	1
1	B	13	60	NE	1
1	B	14	35	NE	1
1	B	15	9	E	2
1	B	16	31	N	1
1	B	17	17	NE	2
1	B	18	17	NE	1
1	B	19	50	NE	1
1	B	20	23	E	1
1	B	21	16	NW	3
1	B	22	10	NW	1
1	B	23	90	NE	1
1	B	24	3	E	1
1	B	25	23	S	1
1	B	26	6	S	1
1	B	27	172	SE	1

1	B	28	29	S	1
1	B	29	NA	NA	1
1	B	1	10	SE	1
1	B	2	10	S	2
1	B	3	7	E	1
1	B	4	107	NW	1
1	B	5	4	S	2
1	B	6	NA	NA	1
1	B	1	35	SW	1
1	B	2	5	N	2
1	B	3	9	N	1
1	B	4	50	SE	1
1	B	5	19	SW	1
1	B	6	21	SW	1
1	B	7	7	E	2
1	B	8	NA	NA	1
1	B	1	27	S	1
1	B	2	175	SW	4
1	B	3	39	S	2
1	B	4	24	SE	1
1	B	5	55	SW	1
1	B	6	NA	NA	1
1	B	1	16	S	1
1	B	2	26	S	2
1	B	3	97	S	1
1	B	4	NA	NA	1
1	B	1	9	N	2
1	B	2	224	NE	1
1	B	3	204	SE	3
1	B	4	3	N	6
1	B	5	NA	NA	2
1	B	1	13	N	1
1	B	2	NA	NA	2
1	B	1	31	SE	1
1	B	2	25	S	1
1	B	3	136	SE	1
1	B	4	257	S	2
1	B	5	218	SE	1
1	B	6	9	S	2
1	B	7	240	NE	1
1	B	8	NA	NA	1
1	B	1	86	N	1
1	B	2	6	E	1

1	B	3	24	E	2
1	B	4	14	E	1
1	B	5	5	SE	3
1	B	6	236	SE	2
1	B	7	60	E	1
1	B	8	42	NE	1
1	B	9	49	NE	1
1	B	10	15	NE	1
1	B	11	3	W	2
1	B	12	27	NE	1
1	B	13	5	N	1
1	B	14	29	NE	1
1	B	15	2	SW	1
1	B	16	11	NE	1
1	B	17	23	E	1
1	B	18	60	E	1
1	B	19	72	NE	2
1	B	20	35	NE	2
1	B	21	170	N	3
1	B	22	23	NW	3
1	B	23	6	N	1
1	B	24	13	W	1
1	B	25	5	E	1
1	B	26	NA	NA	1
1	B	1	6	N	1
1	B	2	5	N	2
1	B	3	9	E	1
1	B	4	11	E	1
1	B	5	10	E	2
1	B	6	351	SW	2
1	B	7	5	E	2
1	B	8	18	S	1
1	B	9	10	E	2
1	B	10	3	S	4
1	B	11	347	S	2
1	B	12	11	N	1
1	B	13	NA	NA	2
1	B	1	31	SE	1
1	B	2	21	E	3
1	B	3	11	NE	5
1	B	4	152	NE	2
1	B	5	118	NE	2
1	B	6	161	N	2

1	B	7	125	NE	2
1	B	8	81	NW	2
1	B	9	10	W	1
1	B	10	NA	NA	3
1	B	1	170	SE	1
1	B	2	NA	NA	1
1	B	1	28	E	2
1	B	2	NA	NA	1
1	B	1	15	SW	3
1	B	2	10	W	4
1	B	3	NA	NA	2
1	B	1	3	W	1
1	B	2	15	SE	2
1	B	3	45	E	2
1	B	4	15	E	3
1	B	5	NA	NA	1
1	B	1	9	E	6
1	B	2	234	NE	2
1	B	3	61	NW	2
1	B	4	11	NE	3
1	B	5	30	N	1
1	B	6	93	N	2
1	B	7	77	N	1
1	B	8	61	NW	3
1	B	9	153	SW	2
1	B	10	22	S	1
1	B	11	49	S	1
1	B	12	40	W	3
1	B	13	1	SE	1
1	B	14	85	S	2
1	B	15	4	NE	2
1	B	16	11	N	1
1	B	17	45	NE	1
1	B	18	13	SW	1
1	B	19	6	S	1
1	B	20	NA	NA	2
1	B	1	4	NW	2
1	B	2	10	W	4
1	B	3	101	N	4
1	B	4	22	NE	4
1	B	5	23	SE	3
1	B	6	15	W	1
1	B	7	15	S	3

1	B	8	200	SE	3
1	B	9	14	S	2
1	B	10	23	S	1
1	B	11	1	S	3
1	B	12	51	E	1
1	B	13	2	W	2
1	B	14	16	S	2
1	B	15	30	S	3
1	B	16	14	S	1
1	B	17	10	W	3
1	B	18	8	W	2
1	B	19	8	W	5
1	B	20	4	N	2
1	B	21	14	E	2
1	B	22	13	E	1
1	B	23	13	E	3
1	B	24	15	E	5
1	B	25	6	N	1
1	B	26	5	N	1
1	B	27	69	NE	1
1	B	28	36	S	4
1	B	29	15	W	4
1	B	30	45	SW	2
1	B	31	19	W	4
1	B	32	118	W	4
1	B	33	9	N	2
1	B	34	3	NE	6
1	B	35	39	W	2
1	B	36	127	SE	1
1	B	37	110	W	2
1	B	38	6	W	4
1	B	39	53	SW	1
1	B	40	45	SE	1
1	B	41	4	S	1
1	B	42	13	E	3
1	B	43	NA	NA	2
1	B	1	36	S	4
1	B	2	10	E	1
1	B	3	18	E	4
1	B	4	15	SE	1
1	B	5	26	NW	2
1	B	6	19	NW	4
1	B	7	22	NW	2

1	B	8	35	S	3
1	B	9	147	N	2
1	B	10	50	NE	2
1	B	11	NA	NA	1
1	B	1	22	NE	2
1	B	2	30	NE	5
1	B	3	4	NE	4
1	B	4	16	N	1
1	B	5	42	W	2
1	B	6	9	N	1
1	B	7	20	W	5
1	B	8	76	S	2
1	B	9	11	N	1
1	B	10	5	SE	2
1	B	11	7	E	5
1	B	12	20	NE	2
1	B	13	12	W	2
1	B	14	25	NW	4
1	B	15	10	W	3
1	B	16	6	E	4
1	B	17	NA	NA	3
1	B	1	9	E	1
1	B	2	NA	NA	2
1	B	1	12	E	2
1	B	2	10	NE	2
1	B	3	25	NE	2
1	B	4	6	NE	1
1	B	5	125	S	1
1	B	6	79	E	1
1	B	7	5	W	2
1	B	8	12	NE	1
1	B	9	13	NE	2
1	B	10	13	SW	1
1	B	11	14	S	2
1	B	12	27	N	2
1	B	13	6	S	1
1	B	14	89	S	1
1	B	15	20	SW	2
1	B	16	1	N	2
1	B	17	19	E	1
1	B	18	3	N	2
1	B	19	13	SE	1
1	B	20	36	N	1

1	B	21	10	NE	7
1	B	22	25	E	2
1	B	23	116	SE	1
1	B	24	3	N	2
1	B	25	3	W	1
1	B	26	NA	NA	2
1	B	1	193	SE	2
1	B	2	105	SE	1
1	B	3	21	W	1
1	B	4	NA	NA	1
1	B	1	10	E	1
1	B	2	4	S	1
1	B	3	97	NE	1
1	B	4	5	S	1
1	B	5	15	S	2
1	B	6	3	S	1
1	B	7	128	E	1
1	B	8	220	NE	1
1	B	9	420	NW	1
1	B	10	89	W	1
1	B	11	13	SW	1
1	B	12	10	SE	2
1	B	13	5	SE	1
1	B	14	NA	NA	1
1	B	1	31	W	1
1	B	2	45	SW	1
1	B	3	35	NW	2
1	B	4	35	W	2
1	B	5	7	W	2
1	B	6	11	NW	2
1	B	7	139	SE	1
1	B	8	8	W	1
1	B	9	102	NE	2
1	B	10	20	NE	2
1	B	11	34	SE	1
1	B	12	19	W	1
1	B	13	55	SE	1
1	B	14	4	W	1
1	B	15	17	S	NA
1	B	16	38	S	1
1	B	17	32	W	1
1	B	18	205	NE	1
1	B	19	37	N	2

1	B	20	85	NE	1
1	B	21	111	NE	2
1	B	22	18	N	NA
1	B	23	15	N	NA
1	B	24	15	E	NA
1	B	25	22	E	1
1	B	26	12	NE	1
1	B	27	30	W	1
1	B	28	11	N	1
1	B	29	88	S	1
1	B	30	13	S	3
1	B	31	1	W	1
1	B	32	23	SW	2
1	B	33	NA	NA	1
1	B	1	133	E	2
1	B	2	4	W	1
1	B	3	26	N	1
1	B	4	68	N	2
1	B	5	10	W	1
1	B	6	32	NW	1
1	B	7	20	S	2
1	B	8	220	NE	1
1	B	9	40	NE	1
1	B	10	2	E	6
1	B	11	4	N	3
1	B	12	15	N	4
1	B	13	37	SE	1
1	B	14	74	SE	2
1	B	15	10	N	NA
1	B	16	4	N	1
1	B	17	NA	NA	2
1	HB	1	7	SW	1
1	HB	2	39	NE	1
1	HB	3	2.5	NW	2
1	HB	4	27	NW	1
1	HB	5	12	SW	1
1	HB	6	59	NW	1
1	HB	7	60	E	1
1	HB	8	110	S	1
1	HB	9	14	SE	1
1	HB	10	64	SE	2
1	HB	11	114	S	1
1	HB	12	NA	NA	1

1	HB	1	11	W	3
1	HB	2	5	SE	2
1	HB	3	7	S	2
1	HB	4	10	NW	1
1	HB	5	11	SE	2
1	HB	6	22	S	3
1	HB	7	35	S	3
1	HB	8	NA	NA	3
1	HB	1	8	W	NA
1	HB	2	55	SE	NA
1	HB	3	100	S	NA
1	HB	4	80	SW	1
1	HB	5	28	NW	3
1	HB	6	10	W	4
1	HB	7	80	SW	5
1	HB	8	50	W	2
1	HB	9	NA	NA	3
1	HB	1	16	SE	6
1	HB	2	25	NE	3
1	HB	3	251	S	1
1	HB	4	NA	NA	1
1	HB	1	34	NW	1
1	HB	2	367	SW	1
1	HB	3	54	S	2
1	HB	4	15	W	4
1	HB	5	34	SW	1
1	HB	6	23	SE	14
1	HB	7	1	N	3
1	HB	8	21	W	3
1	HB	9	25	SW	2
1	HB	10	16	E	5
1	HB	11	8	SE	1
1	HB	12	21	SW	2
1	HB	13	30	SE	1
1	HB	14	14	W	4
1	HB	15	21	E	1
1	HB	16	NA	NA	3
1	HB	1	1	S	1
1	HB	2	12	N	2
1	HB	3	NA	NA	1
1	HB	1	27	W	7
1	HB	2	4	W	6
1	HB	3	30	S	1

1	HB	4	4	S	1
1	HB	5	13	NW	4
1	HB	6	2	E	1
1	HB	7	40	SW	1
1	HB	8	8	W	7
1	HB	9	5	SW	1
1	HB	10	11	E	7
1	HB	11	29	S	2
1	HB	12	26	SE	3
1	HB	13	4	E	2
1	HB	14	15	W	2
1	HB	15	21	NE	2
1	HB	16	13	NW	6
1	HB	17	72	NW	3
1	HB	18	3	W	5
1	HB	19	1	W	2
1	HB	20	NA	NA	1
1	HB	1	7	N	1
1	HB	2	84	SE	1
1	HB	3	48	SE	2
1	HB	4	55	W	2
1	HB	5	NA	NA	1
1	HB	1	58	SW	1
1	HB	2	12	N	3
1	HB	3	3	S	3
1	HB	4	11	NW	2
1	HB	5	18	W	5
1	HB	6	20	NE	5
1	HB	7	NA	NA	3
1	HB	1	247	W	1
1	HB	2	24	W	1
1	HB	3	29	NW	1
1	HB	4	49	NW	2
1	HB	5	17	S	1
1	HB	6	35	S	1
1	HB	7	142	NW	1
1	HB	8	13	S	2
1	HB	9	13	E	3
1	HB	10	NA	NA	1
1	HB	1	1	W	2
1	HB	2	23	SW	2
1	HB	3	128	SW	1
1	HB	4	5	W	5

1	HB	5	4	S	7
1	HB	6	4	NE	1
1	HB	7	46	S	1
1	HB	8	21	SW	4
1	HB	9	10	W	2
1	HB	10	72	S	2
1	HB	11	1	S	3
1	HB	12	1	N	5
1	HB	13	310	SE	4
1	HB	14	NA	NA	1
1	HB	1	11	SW	1
1	HB	2	10	SW	2
1	HB	3	42	SE	1
1	HB	4	NA	NA	1
1	HB	1	5	S	2
1	HB	2	15	N	1
1	HB	3	5	NE	2
1	HB	4	46	SW	1
1	HB	5	NA	NA	4
1	HB	1	40	NE	1
1	HB	2	15	NE	4
1	HB	3	19	N	2
1	HB	4	5	N	3
1	HB	5	NA	NA	3
1	HB	1	2	E	1
1	HB	2	2	W	10
1	HB	3	NA	NA	6
1	HB	1	4	N	13
1	HB	2	2	E	6
1	HB	3	19	E	4
1	HB	4	1	W	4
1	HB	5	NA	NA	1
1	HB	1	14	W	4
1	HB	2	5	W	3
1	HB	3	48	W	4
1	HB	4	12	SE	3
1	HB	5	NA	NA	1
1	HB	1	35	N	1
1	HB	2	NA	NA	1
1	HB	1	53	S	5
1	HB	2	2	N	6
1	HB	3	6	N	2
1	HB	4	10	W	2

1	HB	5	44	N	3
1	HB	6	12	S	5
1	HB	7	4	W	2
1	HB	8	27	N	3
1	HB	9	NA	NA	2
1	HB	1	18	NW	1
1	HB	2	NA	NA	2
1	HB	1	59	NW	3
1	HB	2	5	N	3
1	HB	3	41	W	1
1	HB	4	5	NW	16
1	HB	5	2	SW	3
1	HB	6	7	SW	6
1	HB	7	NA	NA	4
1	HB	1	30	S	2
1	HB	2	13	SW	2
1	HB	3	NA	NA	2
1	HB	1	213	N	2
1	HB	2	NA	NA	2
1	HB	1	5	S	1
1	HB	2	NA	NA	1
1	HB	1	37	NW	1
1	HB	2	16	W	3
1	HB	3	NA	NA	1
1	HB	4	NA	NA	2
1	HB	5	NA	NA	1
1	HB	1	35	NW	1
1	HB	2	20	S	1
1	HB	3	NA	NA	1
1	HB	1	14	SE	4
1	HB	2	10	E	3
1	HB	3	4	SE	1
1	HB	4	NA	NA	1
1	HB	1	3	W	1
1	HB	2	15	N	2
1	HB	3	NA	NA	1
1	HB	1	30	W	2
1	HB	2	99	S	1
1	HB	3	2	S	3
1	HB	4	28	S	2
1	HB	5	9	S	2
1	HB	6	9	SW	1
1	HB	7	46	W	2

1	HB	8	NA	NA	1
1	HB	1	2	E	3
1	HB	2	NA	NA	2
1	HB	1	28	W	2
1	HB	2	NA	NA	1
1	HB	1	24	SW	1
1	HB	2	197	SW	2
1	HB	3	60	SE	2
1	HB	4	NA	NA	1
1	HB	1	9	S	2
1	HB	2	5	E	2
1	HB	3	15	SW	1
1	HB	4	22	S	1
1	HB	5	4	S	2
1	HB	6	177	NE	1
1	HB	7	198	SW	1
1	HB	8	75	NW	1
1	HB	9	16	N	3
1	HB	10	36	SE	1
1	HB	11	183	NE	1
1	HB	12	1	S	1
1	HB	13	8	S	1
1	HB	14	43	SW	2
1	HB	15	4	S	2
1	HB	16	318	S	1
1	HB	17	51	S	1
1	HB	18	169	S	1
1	HB	19	10	W	1
1	HB	20	115	NW	1
1	HB	21	48	W	1
1	HB	22	46	NW	1
1	HB	23	NA	NA	1
1	HB	1	20	NW	3
1	HB	2	5	W	1
1	HB	3	24	NW	3
1	HB	4	150	NE	2
1	HB	5	37	S	8
1	HB	6	NA	NA	4
1	B	1	174	S	2
1	B	2	NA	NA	1
1	HB	1	38	SE	5
1	HB	2	283	E	2
1	HB	3	585	E	2

1	HB	4	NA	NA	3
1	HB	1	4	N	1
1	HB	2	NA	NA	1
1	HB	1	24	N	2
1	HB	2	NA	NA	1
1	HB	1	49	SW	3
1	HB	2	13	SW	1
1	HB	3	6	SW	1
1	HB	4	125	NW	1
1	HB	5	256	S	1
1	HB	6	138	N	1
1	HB	7	105	NE	1
1	HB	8	89	NE	2
1	HB	9	16	N	2
1	HB	10	NA	NA	2
1	HB	1	196	SE	1
1	HB	2	257	SE	2
1	HB	3	NA	NA	2
1	HB	1	149	N	1
1	HB	2	NA	NA	1
1	HB	1	20	NE	3
1	HB	2	3	E	2
1	HB	3	NA	NA	2
1	LC	1	5	SE	5
1	LC	2	16	NE	7
1	LC	3	NA	NA	2
1	LC	1	25	W	1
1	LC	2	NA	NA	2
1	LC	1	39	SW	1
1	LC	2	NA	NA	1
1	LC	3	NA	NA	1
1	LC	4	39	SW	1
1	LC	5	NA	NA	4
1	LC	1	10	E	2
1	LC	2	16	NE	2
1	LC	3	NA	NA	1
1	LC	1	23	W	3
1	LC	2	20	N	2
1	LC	3	NA	NA	4
1	LC	1	34	N	1
1	LC	2	41	S	1
1	LC	3	33	NE	3
1	LC	4	109	SE	4

1	LC	5	2	NW	2
1	LC	6	NA	NA	1
1	LC	1	131	NW	2
1	LC	2	10	N	1
1	LC	3	10	W	3
1	LC	4	19	SW	1
1	LC	5	NA	NA	3
1	LC	1	3	S	4
1	LC	2	3	N	2
1	LC	3	15	N	3
1	LC	4	NA	NA	2
1	LC	1	4	N	2
1	LC	2	NA	NA	2
1	LC	1	46	SE	3
1	LC	2	20	S	2
1	LC	3	23	SE	1
1	LC	4	20	SE	1
1	LC	5	NA	NA	1
1	LC	1	10	NE	1
1	LC	2	NA	NA	1
1	LC	1	38	NE	1
1	LC	2	NA	NA	1
1	LC	1	146	W	1
1	LC	2	NA	NA	1
1	LC	1	31	NE	4
1	LC	2	85	N	3
1	LC	3	32	NW	1
1	LC	4	9	NE	3
1	LC	5	16	NE	1
1	LC	6	60	NW	3
1	LC	7	NA	NA	1
1	LC	1	82	SE	1
1	LC	2	30	SE	1
1	LC	3	NA	NA	1
1	LC	1	4	E	1
1	LC	2	NA	NA	1
1	LC	1	94	SW	3
1	LC	2	NA	NA	5
1	LC	1	71	NE	3
1	LC	2	4	N	1
1	LC	3	23	S	2
1	LC	4	22	S	1
1	LC	5	19	E	2

1	LC	6	7	N	2
1	LC	7	NA	NA	2
1	LC	1	3	S	4
1	LC	2	NA	NA	2
1	LC	1	150	NE	2
1	LC	2	14	SW	4
1	LC	3	NA	NA	1
1	LC	1	26	SE	1
1	LC	2	19	SW	1
1	LC	3	NA	NA	1
1	LC	1	3	NE	6
1	LC	2	11	SW	2
1	LC	3	24	SW	2
1	LC	4	NA	NA	1
1	LC	1	3	E	2
1	LC	2	NA	NA	1
1	LC	1	10	NE	2
1	LC	2	20	NE	2
1	LC	3	NA	NA	1
1	LC	1	106	S	1
1	LC	2	NA	NA	2
1	LC	1	48	NE	1
1	LC	2	NA	NA	1
1	LC	1	2	E	1
1	LC	2	20	W	1
1	LC	3	NA	NA	1
1	LC	1	2	W	1
1	LC	2	17	N	4
1	LC	3	15	NE	2
1	LC	4	NA	NA	1
1	LC	1	14	NE	1
1	LC	2	25	NE	1
1	LC	3	10	NE	1
1	LC	4	NA	NA	1
1	LC	1	67	N	1
1	LC	2	5	S	6
1	LC	3	NA	NA	7
1	LC	1	130	NW	2
1	LC	2	15	S	2
1	LC	3	46	NW	10
1	LC	4	12	NW	1
1	LC	5	13	S	1
1	LC	6	32	NW	4

1	LC	7	227	N	1
1	LC	8	NA	NA	1
1	LC	1	16	W	1
1	LC	2	21	E	1
1	LC	3	NA	NA	1
1	LC	1	25	SE	3
1	LC	2	72	NE	2
1	LC	3	NA	NA	1
1	LC	1	14	NE	1
1	LC	2	5	E	1
1	LC	3	NA	NA	1
1	LC	1	75	NE	3
1	LC	2	34	N	1
1	LC	3	13	S	1
1	LC	4	NA	NA	1
1	LC	1	212	NW	1
1	LC	2	NA	NA	1
1	LC	1	20	N	NA
1	LC	2	NA	NA	2
1	B	1	10	W	2
1	B	2	63	W	1
1	B	3	45	NW	1
1	B	4	NA	NA	1
1	B	1	12	S	6
1	B	2	16	S	2
1	B	3	23	NW	1
1	B	4	44	SW	3
1	B	5	23	E	1
1	B	6	29	E	4
1	B	7	170	W	3
1	B	8	104	SE	8
1	B	9	40	SW	2
1	B	10	184	E	3
1	B	11	335	S	1
1	B	12	47	S	2
1	B	13	77	W	2
1	B	14	46	W	3
1	B	15	69	NW	2
1	B	16	223	SW	2
1	B	17	142	SW	2
1	B	18	15	SW	3
1	B	19	30	N	1
1	B	20	37	NE	3

1	B	21	145	SW	2
1	B	22	132	N	2
1	B	23	46	NW	9
1	B	24	74	W	5
1	B	25	NA	NA	1
1	B	1	13	E	4
1	B	2	12	E	1
1	B	3	3	E	2
1	B	4	9	N	5
1	B	5	6	E	1
1	B	6	6	SE	2
1	B	7	11	NE	1
1	B	8	3	E	2
1	B	9	NA	NA	2
2	B	1	6	W	1
2	B	2	3	N	1
2	B	3	11	W	1
2	B	4	11	NE	1
2	B	5	6	SW	1
2	B	6	NA	NA	1
2	B	1	5	W	3
2	B	2	8	N	4
2	B	3	15	NW	1
2	B	4	10	SE	4
2	B	5	7	W	3
2	B	6	4	N	4
2	B	7	27	SW	1
2	B	8	270	NE	3
2	B	9	30	NW	1
2	B	10	2	S	1
2	B	11	73	N	1
2	B	12	56	N	2
2	B	13	11	N	1
2	B	14	115	NW	1
2	B	15	13	NW	1
2	B	16	6	W	1
2	B	17	118	SW	1
2	B	18	NA	NA	1
2	B	1	1	SW	1
2	B	2	19	SW	1
2	B	3	33	N	2
2	B	4	NA	NA	2
2	B	1	2	NW	1

2	B	2	NA	NA	1
2	B	1	10	N	1
2	B	2	19	W	2
2	B	3	17	W	1
2	B	4	8	N	4
2	B	5	17	W	1
2	B	6	20	E	1
2	B	7	7	N	1
2	B	8	4	W	1
2	B	9	37	NW	1
2	B	10	16	N	1
2	B	11	18	N	1
2	B	12	39	SE	1
2	B	13	16	NW	1
2	B	14	16	N	1
2	B	15	10	E	1
2	B	16	132	N	1
2	B	17	46	NE	1
2	B	18	100	NW	1
2	B	19	NA	NA	1
2	B	1	11	SW	2
2	B	2	15	S	3
2	B	3	82	W	1
2	B	4	320	E	1
2	B	5	NA	NA	1
2	B	1	8	E	2
2	B	2	52	SE	1
2	B	3	14	SE	2
2	B	4	4	N	1
2	B	5	209	S	2
2	B	6	162	SE	1
2	B	7	240	SE	1
2	B	8	7	S	1
2	B	9	22	SW	1
2	B	10	26	SW	3
2	B	11	11	W	1
2	B	12	NA	NA	2
2	B	1	18	SW	1
2	B	2	17	S	5
2	B	3	2	NW	3
2	B	4	17	W	1
2	B	5	5	S	4
2	B	6	254	NW	1

2	B	7	NA	NA	2
2	B	1	5	E	3
2	B	2	NA	NA	4
2	B	1	38	N	5
2	B	2	45	S	3
2	B	3	10	SW	1
2	B	4	174	E	1
2	B	5	14	S	1
2	B	6	5	W	1
2	B	7	7	S	1
2	B	8	NA	NA	1
2	B	1	7	S	1
2	B	2	6	S	1
2	B	3	7	S	2
2	B	4	17	SE	3
2	B	5	9	SE	1
2	B	6	20	W	2
2	B	7	7	SW	1
2	B	8	266	W	1
2	B	9	49	SE	1
2	B	10	37	W	1
2	B	11	77	S	1
2	B	12	10	W	1
2	B	13	NA	NA	1
2	B	14	NA	NA	1
2	B	15	NA	NA	1
2	B	16	NA	NA	2
2	B	17	9	E	1
2	B	18	10	S	1
2	B	19	25	S	1
2	B	20	10	S	1
2	B	21	NA	NA	2
2	B	1	20	N	1
2	B	2	8	N	2
2	B	3	350	NE	2
2	B	4	7	S	2
2	B	5	3	N	5
2	B	6	17	W	1
2	B	7	38	E	1
2	B	8	187	NW	1
2	B	9	4	S	1
2	B	10	41	N	1
2	B	11	126	NW	3

2	B	12	NA	NA	1
2	B	1	18	E	1
2	B	2	115	W	1
2	B	3	29	S	1
2	B	4	9	S	1
2	B	5	81	SW	1
2	B	6	6	W	1
2	B	7	2	E	2
2	B	8	7	E	1
2	B	9	NA	NA	1
2	B	1	3	W	4
2	B	2	NA	NA	1
2	B	1	35	SE	1
2	B	2	13	W	2
2	B	3	24	W	2
2	B	4	12	S	1
2	B	5	12	S	2
2	B	6	25	S	1
2	B	7	NA	NA	2
2	B	1	48	E	2
2	B	2	24	W	1
2	B	3	NA	NA	1
2	B	1	6	S	5
2	B	2	4	W	1
2	B	3	21	E	1
2	B	4	4	S	2
2	B	5	14	S	1
2	B	6	7	S	1
2	B	7	21	N	2
2	B	8	3	SW	1
2	B	9	13	NW	4
2	B	10	13	S	1
2	B	11	7	N	2
2	B	12	34	NE	2
2	B	13	4	NE	3
2	B	14	5	N	4
2	B	15	6	N	6
2	B	16	6	S	1
2	B	17	5	W	1
2	B	18	6	W	1
2	B	19	27	E	1
2	B	20	17	N	4
2	B	21	12	W	1

2	B	22	180	S	1
2	B	23	8	S	1
2	B	24	NA	NA	1
2	B	1	52	E	5
2	B	2	26	S	3
2	B	3	225	S	1
2	B	4	10	S	1
2	B	5	4	W	2
2	B	6	9	S	1
2	B	7	23	S	1
2	B	8	20	S	1
2	B	9	NA	NA	1
2	B	1	2	E	1
2	B	2	25	E	1
2	B	3	16	SW	2
2	B	4	61	E	2
2	B	5	71	E	1
2	B	6	10	S	1
2	B	7	96	S	1
2	B	8	10	S	4
2	B	9	NA	NA	8
2	B	10	NA	NA	1
2	B	11	NA	NA	1
2	B	12	6	E	3
2	B	13	3	S	2
2	B	14	62	S	1
2	B	15	8	NE	3
2	B	16	3	N	3
2	B	17	8	SW	2
2	B	18	37	N	3
2	B	19	15	S	2
2	B	20	28	E	1
2	B	21	20	SW	3
2	B	22	31	SE	2
2	B	23	83	NW	5
2	B	24	33	E	1
2	B	25	9	S	1
2	B	26	49	N	1
2	B	27	64	N	2
2	B	28	137	N	1
2	B	29	6	SW	2
2	B	30	130	SW	1
2	B	31	70	S	1

2	B	32	33	W	3
2	B	33	3	S	2
2	B	34	NA	NA	2
2	B	1	125	S	1
2	B	2	34	NE	1
2	B	3	11	E	1
2	B	4	27	N	1
2	B	5	11	N	1
2	B	6	NA	NA	1
2	B	1	45	E	1
2	B	2	167	NW	2
2	B	3	14	NW	5
2	B	4	119	N	1
2	B	5	37	W	1
2	B	6	76	W	1
2	B	7	153	SW	2
2	B	8	NA	NA	1
2	B	1	388	N	1
2	B	2	100	N	2
2	B	3	35	NW	1
2	B	4	NA	NA	1
2	B	1	2	S	2
2	B	2	NA	NA	2
2	B	1	21	N	1
2	B	2	NA	NA	2
2	B	1	3	N	3
2	B	2	3	W	1
2	B	3	40	SE	1
2	B	4	8	N	1
2	B	5	6	N	1
2	B	6	NA	NA	1
2	B	1	17	NW	2
2	B	2	13	N	1
2	B	3	5	E	1
2	B	4	21	E	1
2	B	5	15	NE	1
2	B	6	20	N	1
2	B	7	13	W	1
2	B	8	27	NE	2
2	B	9	6	SW	2
2	B	10	8	SE	2
2	B	11	36	SW	1
2	B	12	14	S	1

2	B	13	85	S	1
2	B	14	17	W	1
2	B	15	6	NE	1
2	B	16	14	SW	1
2	B	17	9	N	1
2	B	18	11	NW	1
2	B	19	10	NE	1
2	B	20	5	W	1
2	B	21	18	N	2
2	B	22	10	N	1
2	B	23	126	SW	1
2	B	24	52	S	2
2	B	25	14	E	1
2	B	26	10	N	2
2	B	27	20	E	1
2	B	28	7	SE	1
2	B	29	3	S	1
2	B	30	19	S	1
2	B	31	NA	NA	1
2	B	1	8	SE	1
2	B	2	197	NE	3
2	B	3	NA	NA	2
2	B	1	17	W	2
2	B	2	10	NW	1
2	B	3	7	W	1
2	B	4	22	SW	1
2	B	5	10	SE	1
2	B	6	4	SW	1
2	B	7	5	S	1
2	B	8	16	NW	1
2	B	9	NA	NA	1
2	B	10	NA	NA	1
2	B	11	NA	NA	1
2	B	12	56	W	1
2	B	13	10	S	3
2	B	14	18	N	1
2	B	15	72	NW	1
2	B	16	9	NW	1
2	B	17	4	NW	1
2	B	18	21	E	1
2	B	19	NA	NA	1
2	B	1	5	S	1
2	B	2	25	NE	1

2	B	3	26	SE	3
2	B	4	25	E	1
2	B	5	15	W	1
2	B	6	9	E	1
2	B	7	25	N	1
2	B	8	12	N	1
2	B	9	12	E	1
2	B	10	6	S	2
2	B	11	5	W	1
2	B	12	10	W	1
2	B	13	9	SE	2
2	B	14	23	S	2
2	B	15	15	NW	1
2	B	16	38	N	2
2	B	17	3	W	2
2	B	18	5	W	1
2	B	19	3	N	1
2	B	20	3	SW	1
2	B	21	22	NW	1
2	B	22	16	S	1
2	B	23	28	W	1
2	B	24	19	N	1
2	B	25	15	E	1
2	B	26	172	NE	1
2	B	27	26	E	1
2	B	28	11	E	1
2	B	29	64	SE	1
2	B	30	4	NE	2
2	B	31	3	SW	2
2	B	32	6	NW	1
2	B	33	11	NE	1
2	B	34	153	S	1
2	B	35	10	S	4
2	B	36	33	SE	1
2	B	37	NA	NA	1
2	B	38	NA	NA	1
2	B	39	12	SE	2
2	B	40	12	S	1
2	B	41	7	S	2
2	B	42	25	E	1
2	B	43	38	SW	1
2	B	44	7	N	2
2	B	45	8	SE	1

2	B	46	39	NW	1
2	B	47	16	W	2
2	B	48	19	W	2
2	B	49	34	S	1
2	B	50	45	SE	1
2	B	51	5	SE	1
2	B	52	145	W	2
2	B	53	NA	NA	1
2	B	1	20	S	1
2	B	2	100	W	1
2	B	3	7	NW	3
2	B	4	67	W	1
2	B	5	NA	NA	2
2	B	1	34	S	2
2	B	2	264	W	1
2	B	3	5	N	1
2	B	4	199	N	2
2	B	5	39	NE	3
2	B	6	19	E	1
2	B	7	31	SE	1
2	B	8	9	SE	2
2	B	9	16	SE	2
2	B	10	NA	NA	1
2	B	1	7	S	5
2	B	2	33	N	3
2	B	3	NA	NA	2
2	B	1	9	E	2
2	B	2	79	E	3
2	B	3	16	E	3
2	B	4	7	NW	1
2	B	5	27	S	1
2	B	6	82	S	2
2	B	7	14	S	1
2	B	8	6	S	4
2	B	9	16	S	1
2	B	10	4	W	1
2	B	11	3	E	3
2	B	12	3	S	2
2	B	13	11	W	3
2	B	14	9	W	1
2	B	15	6	NW	1
2	B	16	3	NW	1
2	B	17	3	W	1

2	B	18	14	W	1
2	B	19	18	W	1
2	B	20	5	W	1
2	B	21	13	W	2
2	B	22	24	S	1
2	B	23	22	S	3
2	B	24	16	S	1
2	B	25	4	S	1
2	B	26	10	W	1
2	B	27	4	W	1
2	B	28	19	SW	3
2	B	29	9	SW	5
2	B	30	7	W	3
2	B	31	30	W	1
2	B	32	7	NW	6
2	B	33	13	W	1
2	B	34	11	W	1
2	B	35	14	N	1
2	B	36	4	W	3
2	B	37	12	W	1
2	B	38	7	W	2
2	B	39	78	SE	1
2	B	40	5	N	4
2	B	41	5	N	1
2	B	42	98	S	1
2	B	43	13	E	1
2	B	44	37	S	2
2	B	45	8	S	1
2	B	46	13	S	2
2	B	47	12	S	1
2	B	48	100	SW	1
2	B	49	NA	NA	2
2	B	50	NA	NA	1
2	B	51	4	SW	2
2	B	52	20	SW	NA
2	B	53	20	W	NA
2	B	54	NA	NA	NA
2	B	1	9	N	4
2	B	2	6	W	4
2	B	3	NA	NA	4
2	B	1	15	N	2
2	B	2	14	E	1
2	B	3	13	SE	2

2	B	4	30	SE	1
2	B	5	31	N	2
2	B	6	14	S	1
2	B	7	135	NW	1
2	B	8	14	W	3
2	B	9	21	S	1
2	B	10	16	S	2
2	B	11	21	W	1
2	B	12	22	N	1
2	B	13	36	N	3
2	B	14	3	W	1
2	B	15	NA	NA	2
2	B	16	NA	NA	2
2	B	17	8	N	1
2	B	18	17	W	1
2	B	19	62	SW	1
2	B	20	52	NW	2
2	B	21	9	N	1
2	B	22	36	W	4
2	B	23	19	NW	1
2	B	24	6	N	1
2	B	25	14	S	2
2	B	26	19	SE	1
2	B	27	7	E	1
2	B	28	NA	NA	1
2	B	1	30	S	2
2	B	2	30	SE	1
2	B	3	190	E	2
2	B	4	NA	NA	1
2	B	1	10	SW	1
2	B	2	140	E	1
2	B	3	126	SE	1
2	B	4	14	SE	1
2	B	5	14	W	3
2	B	6	6	S	2
2	B	7	NA	NA	2
2	B	1	18	NE	3
2	B	2	NA	NA	2
2	B	1	14	SW	1
2	B	2	14	W	3
2	B	3	21	E	1
2	B	4	73	E	3
2	B	5	3	E	2

2	B	6	NA	NA	3
2	B	1	15	S	1
2	B	2	NA	NA	1
2	B	1	33	E	2
2	B	2	42	W	2
2	B	3	46	S	2
2	B	4	103	S	1
2	B	5	112	S	1
2	B	6	16	S	1
2	B	7	42	S	2
2	B	8	16	W	4
2	B	9	5	N	1
2	B	10	57	S	2
2	B	11	3	S	2
2	B	12	9	S	1
2	B	13	37	W	1
2	B	14	18	W	1
2	B	15	NA	NA	1
2	B	1	5	N	3
2	B	2	17	NW	5
2	B	3	28	E	2
2	B	4	8	SW	4
2	B	5	43	NE	1
2	B	6	260	N	4
2	B	7	2	N	1
2	B	8	NA	NA	2
2	B	1	42	SW	2
2	B	2	10	S	1
2	B	3	37	NW	2
2	B	4	NA	NA	1
2	B	1	8	N	1
2	B	2	228	W	1
2	B	3	74	S	1
2	B	4	150	SW	2
2	B	5	3	W	2
2	B	6	26	SW	1
2	B	7	21	W	1
2	B	8	NA	NA	2
2	B	1	17	NW	1
2	B	2	3	N	1
2	B	3	13	W	1
2	B	4	NA	NA	1
2	B	1	18	N	1

2	B	2	14	N	2
2	B	3	5	N	1
2	B	4	6	N	1
2	B	5	2	E	1
2	B	6	319	SW	2
2	B	7	2	S	3
2	B	8	NA	NA	1
2	B	1	8	S	3
2	B	2	15	W	3
2	B	3	9	N	3
2	B	4	10	NW	3
2	B	5	6	N	1
2	B	6	164	E	3
2	B	7	24	S	1
2	B	8	111	SW	1
2	B	9	43	SW	2
2	B	10	38	SW	1
2	B	11	35	S	2
2	B	12	3	NE	2
2	B	13	10	SW	1
2	B	14	84	SW	1
2	B	15	9	E	5
2	B	16	97	W	1
2	B	17	158	W	3
2	B	18	NA	NA	6
2	B	1	4	E	1
2	B	2	74	E	1
2	B	3	53	SE	1
2	B	4	NA	NA	1
2	B	1	82	S	1
2	B	2	3	S	2
2	B	3	29	S	2
2	B	4	13	S	1
2	B	5	2	W	2
2	B	6	23	N	2
2	B	7	14	SE	1
2	B	8	7	W	4
2	B	9	11	NE	3
2	B	10	9	N	2
2	B	11	22	S	1
2	B	12	6	S	1
2	B	13	5	SW	1
2	B	14	7	S	3

2	B	15	7	E	1
2	B	16	13	S	1
2	B	17	17	NE	1
2	B	18	11	S	1
2	B	19	8	S	1
2	B	20	16	SW	1
2	B	21	20	S	1
2	B	22	7	S	1
2	B	23	22	E	2
2	B	24	31	W	2
2	B	25	1	W	2
2	B	26	23	W	2
2	B	27	NA	NA	1
2	B	28	NA	NA	2
2	B	29	6	N	2
2	B	30	35	NE	2
2	B	31	23	E	2
2	B	32	6	E	2
2	B	33	17	E	1
2	B	34	48	SE	1
2	B	35	55	S	1
2	B	36	61	SW	1
2	B	37	25	SW	1
2	B	38	110	S	2
2	B	39	29	E	1
2	B	40	21	S	1
2	B	41	21	W	2
2	B	42	3	S	4
2	B	43	27	S	1
2	B	44	30	S	1
2	B	45	19	E	1
2	B	46	73	E	2
2	B	47	3	N	1
2	B	48	17	NE	3
2	B	49	33	SE	2
2	B	50	25	S	1
2	B	51	18	W	1
2	B	52	5	W	1
2	B	53	9	S	1
2	B	54	3	W	1
2	B	55	4	W	1
2	B	56	36	W	1
2	B	57	26	W	2

2	B	58	9	S	1
2	B	59	13	E	1
2	B	60	83	E	2
2	B	61	7	N	2
2	B	62	13	N	2
2	B	63	9	W	1
2	B	64	45	W	1
2	B	65	34	SW	3
2	B	66	20	W	1
2	B	67	12	NW	1
2	B	68	44	E	2
2	B	69	19	N	2
2	B	70	18	W	1
2	B	71	NA	NA	1
2	B	1	68	E	1
2	B	2	NA	NA	1
2	B	1	11	S	1
2	B	2	9	E	1
2	B	3	15	N	1
2	B	4	NA	NA	2
2	B	1	5	E	3
2	B	2	16	S	2
2	B	3	1	N	1
2	B	4	13	SE	1
2	B	5	100	NE	1
2	B	6	7	N	1
2	B	7	12	E	1
2	B	8	NA	NA	1
2	B	1	13	N	1
2	B	2	10	N	1
2	B	3	43	NW	2
2	B	4	10	NE	4
2	B	5	12	N	5
2	B	6	NA	NA	1
2	B	1	1	NW	5
2	B	2	NA	NA	2
2	B	1	21	N	2
2	B	2	13	N	3
2	B	3	100	N	1
2	B	4	75	N	1
2	B	5	170	NE	1
2	B	6	7	SE	5
2	B	7	20	SE	3

2	B	8	24	E	1
2	B	9	27	S	1
2	B	10	3	N	3
2	B	11	19	SW	4
2	B	12	269	E	1
2	B	13	81	E	3
2	B	14	223	E	2
2	B	15	80	S	1
2	B	16	13	S	1
2	B	17	27	S	2
2	B	18	NA	NA	2
2	B	1	7	E	4
2	B	2	9	SE	2
2	B	3	39	E	3
2	B	4	7	N	1
2	B	5	11	W	1
2	B	6	NA	NA	1
2	B	1	249	W	1
2	B	2	325	N	1
2	B	3	212	N	1
2	B	4	NA	NA	1
2	B	1	30	W	1
2	B	2	50	N	1
2	B	3	6	N	2
2	B	4	NA	NA	2
2	B	1	83	E	1
2	B	2	30	SE	1
2	B	3	89	SE	1
2	B	4	NA	NA	1
2	B	1	10	NW	1
2	B	2	104	E	1
2	B	3	5	SE	1
2	B	4	5	E	2
2	B	5	5	N	3
2	B	6	27	SE	1
2	B	7	8	E	1
2	B	8	15	N	1
2	B	9	NA	NA	1
2	B	1	10	S	2
2	B	2	70	S	2
2	B	3	50	SE	1
2	B	4	15	S	3
2	B	5	40	W	2

2	B	6	NA	NA	2
2	B	1	25	N	3
2	B	2	125	NE	2
2	B	3	7	NW	1
2	B	4	NA	NA	1
2	B	1	17	E	1
2	B	2	16	SE	1
2	B	3	4	S	1
2	B	4	5	S	1
2	B	5	173	SW	1
2	B	6	303	NW	1
2	B	7	26	N	1
2	B	8	5	N	1
2	B	9	NA	NA	1
2	B	1	3	NW	1
2	B	2	13	N	2
2	B	3	NA	NA	2
2	B	1	10	W	1
2	B	2	20	W	1
2	B	3	20	W	1
2	B	4	9	W	1
2	B	5	85	SW	1
2	B	6	71	W	2
2	B	7	113	S	1
2	B	8	83	W	2
2	B	9	77	W	2
2	B	10	17	W	2
2	B	11	NA	NA	1
2	B	1	29	N	2
2	B	2	20	W	1
2	B	3	NA	NA	1
2	B	1	10	W	1
2	B	2	34	W	2
2	B	3	73	E	2
2	B	4	19	SE	1
2	B	5	24	N	1
2	B	6	49	W	1
2	B	7	11	W	1
2	B	8	30	W	1
2	B	9	NA	NA	1
2	B	1	3	N	2
2	B	2	6	W	1
2	B	3	NA	NA	2

2	B	1	25	E	1
2	B	2	25	E	1
2	B	3	NA	NA	1
2	B	1	25	SE	1
2	B	2	15	SE	3
2	B	3	7	W	1
2	B	4	251	N	1
2	B	5	22	E	1
2	B	6	17	N	1
2	B	7	16	SE	1
2	B	8	34	E	1
2	B	9	35	SE	1
2	B	10	11	N	1
2	B	11	11	NE	1
2	B	12	71	SE	1
2	B	13	6	NW	2
2	B	14	14	SW	1
2	B	15	11	W	1
2	B	16	16	SW	1
2	B	17	16	S	1
2	B	18	15	SW	1
2	B	19	21	S	1
2	B	20	7	SE	1
2	B	21	16	E	1
2	B	22	17	W	NA
2	B	23	16	N	1
2	B	24	164	NE	2
2	B	25	20	SW	1
2	B	26	NA	NA	1
2	HB	1	91	NW	1
2	HB	2	56	SW	1
2	HB	3	8	N	1
2	HB	4	4	N	1
2	HB	5	NA	NA	1
2	HB	1	96	SE	1
2	HB	2	11	S	1
2	HB	3	43	E	1
2	HB	4	20	S	6
2	HB	5	16	N	2
2	HB	6	14	S	2
2	HB	7	45	E	1
2	HB	8	86	W	1
2	HB	9	5	N	2

2	HB	10	8	N	4
2	HB	11	47	SW	2
2	HB	12	43	W	3
2	HB	13	16	N	1
2	HB	14	86	NE	1
2	HB	15	3	NW	14
2	HB	16	7	N	6
2	HB	17	3	W	1
2	HB	18	7	W	1
2	HB	19	4	W	2
2	HB	20	7	W	3
2	HB	21	3	NW	6
2	HB	22	39	N	2
2	HB	23	36	W	1
2	HB	24	82	N	2
2	HB	25	50	S	2
2	HB	26	239	SE	1
2	HB	27	103	E	2
2	HB	28	255	N	1
2	HB	29	15	S	3
2	HB	30	30	N	1
2	HB	31	3	W	2
2	HB	32	18	W	1
2	HB	33	5	N	1
2	HB	34	8	N	4
2	HB	35	10	NE	2
2	HB	36	9	SW	2
2	HB	37	40	E	5
2	HB	38	3	NW	5
2	HB	39	8	NE	1
2	HB	40	13	N	1
2	HB	41	5	NW	2
2	HB	42	6	NE	4
2	HB	43	4	NE	1
2	HB	44	60	E	2
2	HB	45	18	E	2
2	HB	46	10	NE	1
2	HB	47	11	S	3
2	HB	48	32	SE	1
2	HB	49	6	E	5
2	HB	50	3	N	2
2	HB	51	3	W	1
2	HB	52	38	W	1

2	HB	53	44	W	1
2	HB	54	113	W	5
2	HB	55	22	NW	1
2	HB	56	113	N	1
2	HB	57	44	N	1
2	HB	58	301	NA	2
2	HB	59	120	W	1
2	HB	60	13	NW	1
2	HB	61	72	W	1
2	HB	62	70	W	3
2	HB	63	5	N	1
2	HB	64	190	SW	1
2	HB	65	16	S	2
2	HB	66	5	NW	5
2	HB	67	NA	NA	4
2	HB	68	NA	NA	3
2	HB	69	87	W	1
2	HB	70	125	NW	2
2	HB	71	49	N	1
2	HB	72	NA	NA	1
2	HB	1	10	S	4
2	HB	2	8	S	2
2	HB	3	21	S	2
2	HB	4	NA	NA	2
2	HB	1	68	N	1
2	HB	2	45	NE	2
2	HB	3	NA	NA	1
2	HB	1	33	W	2
2	HB	2	NA	NA	2
2	HB	1	3	W	2
2	HB	2	54	NE	1
2	HB	3	14	NW	1
2	HB	4	9	E	1
2	HB	5	50	NW	4
2	HB	6	5	N	1
2	HB	7	NA	NA	1
2	HB	1	5	SE	1
2	HB	2	2	N	1
2	HB	3	4	SE	1
2	HB	4	7	N	1
2	HB	5	6	S	2
2	HB	6	15	N	2
2	HB	7	11	N	2

2	HB	8	NA	NA	2
2	HB	1	395	NE	1
2	HB	2	NA	NA	1
2	HB	1	10	N	1
2	HB	2	3	W	2
2	HB	3	NA	NA	1
2	HB	1	8	W	3
2	HB	2	NA	NA	1
2	HB	1	15	S	3
2	HB	2	18	NW	1
2	HB	3	2	S	1
2	HB	4	11	SE	2
2	HB	5	4	SE	2
2	HB	6	5	S	1
2	HB	7	9	S	1
2	HB	8	39	W	1
2	HB	9	23	SE	2
2	HB	10	10	NE	2
2	HB	11	28	E	1
2	HB	12	49	N	1
2	HB	13	NA	NA	1
2	HB	1	8	W	2
2	HB	2	1	W	2
2	HB	3	NA	NA	1
2	HB	1	11	E	1
2	HB	2	9	N	2
2	HB	3	7	N	2
2	HB	4	22	W	1
2	HB	5	8	NW	2
2	HB	6	2	SW	3
2	HB	7	10	E	1
2	HB	8	15	E	1
2	HB	9	15	S	4
2	HB	10	8	NW	1
2	HB	11	13	NE	5
2	HB	12	11	S	2
2	HB	13	25	SE	3
2	HB	14	10	N	3
2	HB	15	5	S	1
2	HB	16	47	NE	2
2	HB	17	47	NE	2
2	HB	18	5	W	1
2	HB	19	12	NW	1

2	HB	20	15	N	1
2	HB	21	15	N	1
2	HB	22	6	SW	2
2	HB	23	22	E	1
2	HB	24	5	NE	1
2	HB	25	3	S	1
2	HB	26	12	N	1
2	HB	27	70	N	1
2	HB	28	5	E	4
2	HB	29	18	W	7
2	HB	30	9	NW	1
2	HB	31	2	S	1
2	HB	32	6	NW	1
2	HB	33	12	NW	1
2	HB	34	11	W	1
2	HB	35	18	N	1
2	HB	36	20	E	1
2	HB	37	80	N	1
2	HB	38	55	N	1
2	HB	39	NA	NA	1
2	HB	1	11	SE	3
2	HB	2	4	S	2
2	HB	3	9	S	3
2	HB	4	NA	NA	1
2	HB	1	89	E	1
2	HB	2	4	S	3
2	HB	3	3	N	1
2	HB	4	6	S	2
2	HB	5	20	NE	2
2	HB	6	2	NW	1
2	HB	7	31	S	1
2	HB	8	267	W	1
2	HB	9	15	W	2
2	HB	10	12	N	1
2	HB	11	148	W	2
2	HB	12	2	E	1
2	HB	13	131	NW	4
2	HB	14	34	W	1
2	HB	15	32	W	2
2	HB	16	2	SW	1
2	HB	17	15	SW	3
2	HB	18	170	N	1
2	HB	19	NA	NA	1

2	HB	1	24	N	1
2	HB	2	7	N	1
2	HB	3	5	N	2
2	HB	4	13	S	1
2	HB	5	7	W	2
2	HB	6	6	W	2
2	HB	7	2	NW	3
2	HB	8	NA	NA	1
2	HB	1	3	SW	1
2	HB	2	10	E	2
2	HB	3	194	NE	1
2	HB	4	18	SW	1
2	HB	5	19	W	3
2	HB	6	6	W	1
2	HB	7	NA	NA	2
2	HB	1	2	NW	2
2	HB	2	9	N	1
2	HB	3	197	W	1
2	HB	4	20	W	1
2	HB	5	39	W	1
2	HB	6	NA	NA	2
2	HB	1	30	SE	1
2	HB	2	36	S	2
2	HB	3	36	W	1
2	HB	4	17	NW	1
2	HB	5	9	N	1
2	HB	6	20	W	1
2	HB	7	6	W	1
2	HB	8	8	SW	1
2	HB	9	24	S	1
2	HB	10	NA	NA	1
2	HB	1	4	S	2
2	HB	2	1	S	2
2	HB	3	16	N	1
2	HB	4	17	NE	1
2	HB	5	69	E	2
2	HB	6	307	NE	1
2	HB	7	4	W	1
2	HB	8	27	N	1
2	HB	9	NA	NA	2
2	HB	1	2	NW	1
2	HB	2	9	N	1
2	HB	3	NA	NA	2

2	HB	1	176	NE	1
2	HB	2	45	W	1
2	HB	3	6	S	1
2	HB	4	3	N	1
2	HB	5	21	N	1
2	HB	6	5	NE	1
2	HB	7	17	NE	1
2	HB	8	13	W	1
2	HB	9	30	W	2
2	HB	10	5	NW	1
2	HB	11	29	SW	1
2	HB	12	4	SW	1
2	HB	13	11	W	3
2	HB	14	21	S	1
2	HB	15	67	S	2
2	HB	16	8	S	2
2	HB	17	47	SW	1
2	HB	18	NA	NA	1
2	HB	1	7	S	2
2	HB	2	15	W	1
2	HB	3	8	NE	2
2	HB	4	65	E	1
2	HB	5	36	SW	2
2	HB	6	1	E	1
2	HB	7	10	N	1
2	HB	8	4	NW	1
2	HB	9	17	NW	1
2	HB	10	19	N	2
2	HB	11	179	N	2
2	HB	12	14	NW	3
2	HB	13	NA	NA	3
2	HB	1	20	E	2
2	HB	2	11	W	1
2	HB	3	25	W	4
2	HB	4	8	SW	1
2	HB	5	32	SW	1
2	HB	6	12	S	2
2	HB	7	74	E	3
2	HB	8	3	N	1
2	HB	9	20	NW	1
2	HB	10	7	W	6
2	HB	11	14	E	1
2	HB	12	10	SE	1

2	HB	13	21	N	2
2	HB	14	24	NW	1
2	HB	15	44	SE	4
2	HB	16	34	NW	1
2	HB	17	7	E	2
2	HB	18	27	S	1
2	HB	19	12	SW	2
2	HB	20	8	SW	4
2	HB	21	25	W	4
2	HB	22	1	W	2
2	HB	23	1	N	1
2	HB	24	26	NE	2
2	HB	25	14	SW	1
2	HB	26	10	S	1
2	HB	27	NA	NA	1
2	HB	1	92	N	1
2	HB	2	224	W	1
2	HB	3	3	W	1
2	HB	4	3	SW	4
2	HB	5	49	W	1
2	HB	6	15	NW	1
2	HB	7	3	S	1
2	HB	8	6	NW	1
2	HB	9	51	E	4
2	HB	10	2	W	6
2	HB	11	1	S	1
2	HB	12	24	W	1
2	HB	13	26	NE	1
2	HB	14	5	E	1
2	HB	15	5	W	1
2	HB	16	3	N	4
2	HB	17	3	E	1
2	HB	18	2	SW	1
2	HB	19	2	NE	1
2	HB	20	20	NW	1
2	HB	21	13	NE	1
2	HB	22	5	NE	1
2	HB	23	16	S	1
2	HB	24	27	SE	2
2	HB	25	10	S	1
2	HB	26	NA	NA	2
2	HB	1	21	NW	4
2	HB	2	162	W	2

2	HB	3	131	W	3
2	HB	4	6	SE	13
2	HB	5	6	N	2
2	HB	6	110	NW	1
2	HB	7	2	SW	1
2	HB	8	1	NW	2
2	HB	9	3	SW	1
2	HB	10	173	NE	2
2	HB	11	3	N	2
2	HB	12	19	SE	1
2	HB	13	NA	NA	1
2	HB	1	15	N	1
2	HB	2	7	NW	4
2	HB	3	10	N	5
2	HB	4	3	NW	8
2	HB	5	7	N	2
2	HB	6	2	W	1
2	HB	7	27	NW	NA
2	HB	8	3	W	3
2	HB	9	3	SE	3
2	HB	10	5	SE	1
2	HB	11	3	NE	4
2	HB	12	5	E	2
2	HB	13	7	E	1
2	HB	14	3	S	4
2	HB	15	3	N	1
2	HB	16	NA	NA	1
2	HB	1	367	S	1
2	HB	2	21	N	2
2	HB	3	NA	NA	2
2	LC	1	53	S	1
2	LC	2	72	S	12
2	LC	3	14	SW	1
2	LC	4	NA	NA	1
2	LC	1	12	S	1
2	LC	2	38	NW	2
2	LC	3	NA	NA	1
2	LC	1	30	NE	1
2	LC	2	42	N	1
2	LC	3	NA	NA	1
2	LC	1	52	W	1
2	LC	2	10	NE	1
2	LC	3	NA	NA	4

2	LC	1	76	NW	2
2	LC	2	5	SW	1
2	LC	3	152	S	1
2	LC	4	60	S	1
2	LC	5	37	NE	2
2	LC	6	NA	NA	1
2	LC	1	29	S	2
2	LC	2	65	E	1
2	LC	3	NA	NA	1
2	LC	1	6	N	1
2	LC	2	NA	NA	1
2	LC	1	4	W	1
2	LC	2	24	E	2
2	LC	3	NA	NA	1
2	LC	1	4	N	2
2	LC	2	NA	NA	1
2	LC	1	52	SW	1
2	LC	2	NA	NA	1
2	LC	1	20	W	2
2	LC	2	9	W	1
2	LC	3	4	S	1
2	LC	4	44	W	1
2	LC	5	7	N	2
2	LC	6	31	N	2
2	LC	7	8	W	1
2	LC	8	NA	NA	1
2	LC	1	78	W	1
2	LC	2	68	W	2
2	LC	3	NA	NA	1
2	LC	1	9	S	1
2	LC	2	53	E	1
2	LC	3	NA	NA	1
2	LC	1	37	E	3
2	LC	2	1	W	1
2	LC	3	NA	NA	1
2	LC	1	10	SE	1
2	LC	2	2	W	1
2	LC	3	NA	NA	3
2	LC	1	75	NW	2
2	LC	2	42	W	2
2	LC	3	NA	NA	1
2	LC	1	48	W	1
2	LC	2	33	W	2

2	LC	3	NA	NA	1
2	LC	1	35	N	1
2	LC	2	139	W	1
2	LC	3	57	SW	1
2	LC	4	11	SW	1
2	LC	5	27	SW	4
2	LC	6	11	S	1
2	LC	7	42	S	1
2	LC	8	19	W	1
2	LC	9	40	S	1
2	LC	10	38	S	1
2	LC	11	7	W	1
2	LC	12	2	NW	1
2	LC	13	72	SE	2
2	LC	14	NA	NA	1
2	LC	1	11	SW	2
2	LC	2	NA	NA	2
2	LC	1	87	W	1
2	LC	2	42	SW	2
2	LC	3	NA	NA	1
2	LC	1	12	SE	3
2	LC	2	53	E	1
2	LC	3	NA	NA	1
2	LC	1	16	SE	1
2	LC	2	9	E	3
2	LC	3	5	S	16
2	LC	4	154	SE	2
2	LC	5	12	S	1
2	LC	6	NA	NA	6
2	LC	1	55	W	1
2	LC	2	18	SW	1
2	LC	3	22	N	1
2	LC	4	NA	NA	1
2	LC	1	8	N	3
2	LC	2	19	W	1
2	LC	3	47	S	1
2	LC	4	26	SW	1
2	LC	5	10	NW	1
2	LC	6	NA	NA	2
2	LC	1	109	SE	2
2	LC	2	25	SE	2
2	LC	3	NA	NA	1
2	LC	1	4	S	2

2	LC	2	NA	NA	1
2	LC	1	18	SW	1
2	LC	2	NA	NA	2
2	LC	1	8	N	1
2	LC	2	4	NW	7
2	LC	3	90	S	3
2	LC	4	15	W	1
2	LC	5	NA	NA	1
2	LC	1	10	NE	2
2	LC	2	2	N	1
2	LC	3	13	E	3
2	LC	4	3	W	1
2	LC	5	4	NE	3
2	LC	6	25	W	3
2	LC	7	14	NE	2
2	LC	8	15	SW	1
2	LC	9	27	E	3
2	LC	10	16	NE	1
2	LC	11	44	SE	4
2	LC	12	105	NW	2
2	LC	13	11	E	2
2	LC	14	125	S	1
2	LC	15	74	NE	1
2	LC	16	NA	NA	1
2	LC	1	302	SE	2
2	LC	2	NA	NA	1
2	LC	1	6	E	2
2	LC	2	40	S	1
2	LC	3	20	SE	2
2	LC	4	9	N	2
2	LC	5	3	NW	1
2	LC	6	85	W	1
2	LC	7	20	N	1
2	LC	8	8	N	1
2	LC	9	4	N	1
2	LC	10	73	SW	1
2	LC	11	49	N	1
2	LC	12	26	S	1
2	LC	13	171	SE	2
2	LC	14	5	N	1
2	LC	15	5	N	1
2	LC	16	9	NE	2
2	LC	17	7	NW	2

2	LC	18	10	N	1
2	LC	19	NA	NA	1
2	LC	1	39	E	1
2	LC	2	24	E	1
2	LC	3	NA	NA	1
2	LC	1	6	NW	1
2	LC	2	NA	NA	1
2	LC	1	63	SW	1
2	LC	2	64	NE	2
2	LC	3	53	SW	1
2	LC	4	95	W	1
2	LC	5	NA	NA	1
2	LC	1	174	NE	1
2	LC	2	NA	NA	1
2	LC	1	34	N	1
2	LC	2	NA	NA	1
2	LC	1	22	S	2
2	LC	2	NA	NA	1
2	LC	1	54	E	1
2	LC	2	9	NE	4
2	LC	3	46	S	2
2	LC	4	21	SW	1
2	LC	5	100	N	2
2	LC	6	2	E	1
2	LC	7	11	S	2
2	LC	8	13	E	4
2	LC	9	9	SE	4
2	LC	10	8	N	5
2	LC	11	9	E	1
2	LC	12	NA	NA	5
2	LC	1	6	N	1
2	LC	2	51	W	1
2	LC	3	NA	NA	1
2	LC	1	17	E	1
2	LC	2	19	NE	2
2	LC	3	30	S	1
2	LC	4	45	W	2
2	LC	5	35	S	1
2	LC	6	NA	NA	1
2	LC	1	13	W	2
2	LC	2	21	S	1
2	LC	3	24	N	2
2	LC	4	12	SW	1

2	LC	5	NA	NA	1
2	LC	1	30	E	1
2	LC	2	NA	NA	1
2	LC	1	194	NW	1
2	LC	2	NA	NA	1
2	LC	1	3	SW	1
2	LC	2	45	NE	1
2	LC	3	NA	NA	1
2	LC	1	21	W	1
2	LC	2	52	NW	1
2	LC	3	3	W	1
2	LC	4	NA	NA	1
2	LC	1	2	E	1
2	LC	2	13	N	2
2	LC	3	13	N	1
2	LC	4	64	E	2
2	LC	5	32	NE	1
2	LC	6	NA	NA	2
2	LC	1	39	S	1
2	LC	2	NA	NA	1
2	LC	1	8	NW	1
2	LC	2	NA	NA	1
2	LC	1	35	S	1
2	LC	2	35	W	1
2	LC	3	NA	NA	1
2	LC	1	15	E	2
2	LC	2	3	E	1
2	LC	3	13	N	1
2	LC	4	NA	NA	1
2	LC	1	12	NW	1
2	LC	2	NA	NA	1
2	LC	1	17	SW	1
2	LC	2	NA	NA	2
2	LC	1	71	S	1
2	LC	2	103	SW	1
2	LC	3	NA	NA	1

Legend

B	bumble bee	SW	South west
HB	honey bee	S	South
LCB	leafcutting bee	W	West

		SE	south east
NA	not available	E	east
		NW	north west
Dist	Distance	N	north west
Dir	Direction	NE	north east
