# Supplementary material for: Bee species perform distinct foraging behaviors that are best described by different movement models
Source: Sci Rep. 2023 Jan 2;13:71. doi: 10.1038/s41598-022-26858-9 (PMC9807645; doi:10.1038/s41598-022-26858-9)
Supplement: Supplementary file 2 — Supplementary Information 2. [file 41598_2022_26858_MOESM2_ESM.pdf]

Bee species perform distinct foraging behaviors that are best described by different movement models

Johanne Brunet[1], Qi Jiang[2$], Yang Zhao[2+], Margaret W. Thairu[3#] and Murray K. Clayton[2]

[1] Vegetable Crops Research Unit, United States Department of Agriculture-Agricultural Research Service, Madison, WI 53706, United States

[2] Department of Statistics, University of Wisconsin, Madison, WI 53706, USA

[3] Department of Entomology, University of Wisconsin, Madison, WI 53706, USA

$ Current address: Goldman Sachs, 200 West Street, New York, NY 10282

[+] Current address: Gilead Sciences, 333 Lakeside Dr, Foster City, CA 94402

[#] Current address: Department of Bacteriology, University of Wisconsin, Madison, WI.

Corresponding author: Johanne Brunet Johanne.Brunet@usda.gov

Supplementary Fig. S1. The frequency distribution of simulated mean net distances traveled under the Random Distance-Random Direction Model for each bee species each year. The vertical dash line represents the empirical value. The top graphs are for Year 1 for each bee species and the bottom graphs are for Year 2.

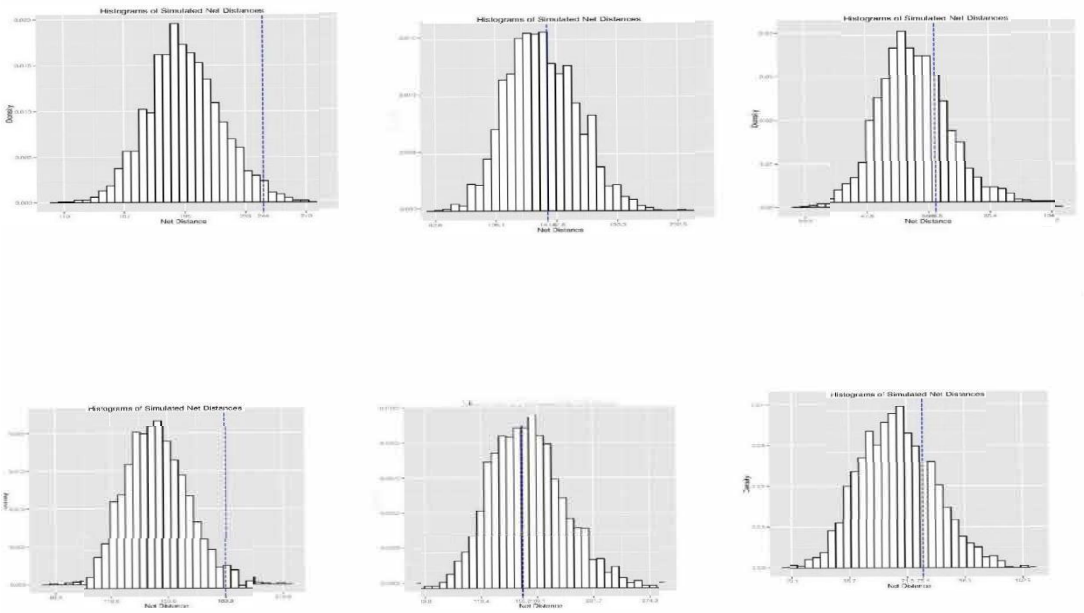

Supplementary Fig. S2. Representative foraging bouts for each of the three bee species.

A) Bumble bees

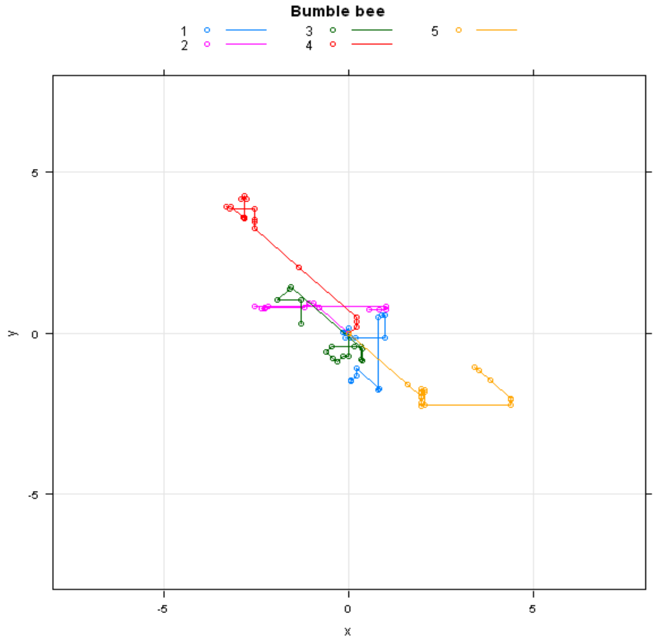

B) Honey bees

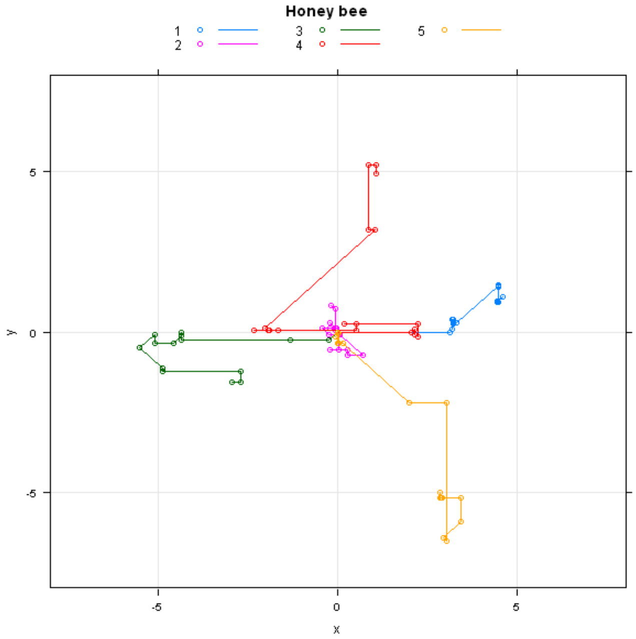

C) Leafcutting bees

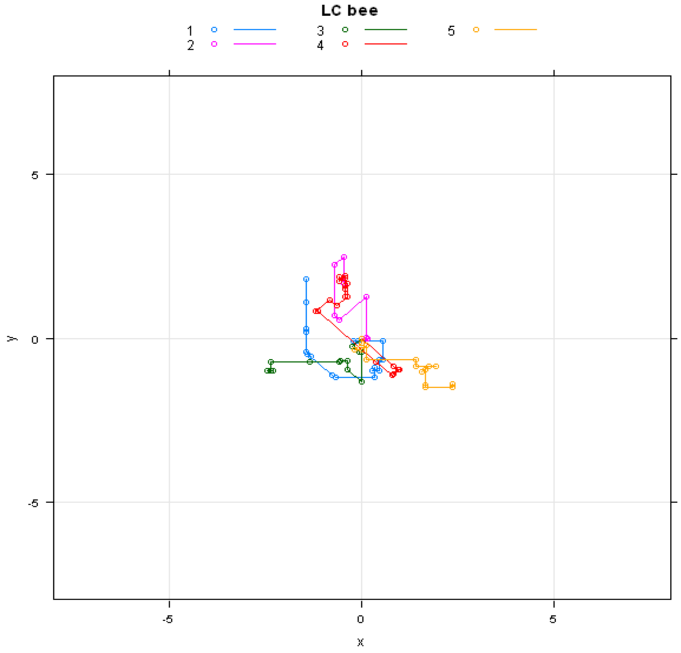

Supplementary Table S2 Distance models over all bee species for year 1, with AIC values. An X indicates that the variable is included in the model. The variables patch and foraging bout are random variables. The variable clip is added either as a random variable, where in combination with random foraging bout, it creates a model that is sometimes called the "compound symmetry" model or as an AR(1) repeated measure structure, described in the text. Significant variables are followed by an asterisk with *: 0.1 ≤ p <0.05; **: 0.001 ≤ p < 0.01.

Bee	Flower	Patch	Foraging bout	AR (1)	Clip	AIC
	X**		X**	X*		3108.9
	X**	X	X**	X*		3108.9
X	X**		X**	X*		3111.2
X	X**	X	X**	X*		3111.2
	X**	X	X**		X	3111.4
	X**	X	X**			3111.4
	X**		X**			3111.4
	X**		X**		X	3111.4
X	X**	X	X**		X	3113.5
X	X**	X	X**			3113.5
X	X**		X**			3113.5
X	X**		X**		X	3113.5
	X**	X		X**		3128.9
	X**			X**		3128.9
X	X**	X		X**		3132.3
X	X**			X**		3132.3
	X**	X				3157.8
	X**	X			X	3157.8
	X**				X	3157.8
X	X**	X				3161
X	X**	X			X	3161
	X**				X	3161

Supplementary Table S3. Distance models over all bee species for year 2, with AIC values. An X indicates that the variable is included in the model. The variables patch and foraging bout are random variables. The variable clip is added either as a random variable, where in combination with random foraging bout, it creates a model that is sometimes called the "compound symmetry" model or as an AR(1) repeated measure structure, described in the text. Significant variables are followed by an asterisk with *: $0.1 \le p < 0.05$; **: $0.001 \le p < 0.01$.

Bee	Flower	Patch	Foraging bout	AR (1)	Clip	AIC
X**	X**		X*	X*		3561.3
X**	X**	X	X*	X*		3561.3
X**	X**	X	X**		X	*3565.7*
X**	X**	X	X**			3565.7
X**	X**		X**			3565.7
X**	X**		X**		X	3565.7
X**	X**	X		X**		3566.9
X**	X**			X**		3566.9
	X**	X	X*	X**		3569.6
	X**	X	X*	X**		3571.6
	X**	X		X**		3574.7
	X**		X**			3574.7
	X**			X**		3574.7
	X**		X**		X	3574.7
	X**	X	X**		X	3576.6
	X**	X	X**			3576.6
X**	X**	X				3580.3
X**	X**	X			X	3580.3
X**	X**				X	3580.3
	X**	X				3591.3
	X**	X			X	3591.3
	X**				X	3591.3

Supplementary Table S4. Distance models for each bee species for year 1, with AIC values. An X indicates that the variable is included in the model. The variables patch and foraging bout are random variables. The variable clip is added either as a random variable, where in combination with random foraging bout, it creates a model that is sometimes called the "compound symmetry" model or as an AR(1) repeated measure structure, described in the text. Significant variables are followed by an asterisk with *: $0.1 \leq p < 0.05$; **: $0.001 \leq p < 0.01$.

Flower	Patch	Foraging bout	AR (1)	Clip	AIC
		Bumble bee			
X**		X**	X*		2252.1
X**	X	X**	X*		2252.1
X**	X	X**			2255.3
X**	X	X**		X	2255.3
X**	X		X**		2282.4
X**	X				2323.2
X**	X			X	2323.2
X**		X**		X	2363.6
X**		X**			2363.6
X**			X**		2395.3
X**				X	2435.8
		Honey bee			
X**	X				689.8
X**	X			X	689.8
X**	X	X	X		691

X**	X	X		X	691
X**				X	691.5
X**	X	X		X	691.7
X**	X	X			691.7
X**				X	691.9
X**		X		X	692.9
X**		X			692.9
X**		X	X		693.9
Leafcutting bee					
X				X	265.5
X	X			X	265.5
X	X				265.5
X	X	X			267.4
X		X			267.4
X	X	X		X	267.4
X		X		X	267.4
X			X		267.5
X	X		X		267.5
X		X	X		269.4
X	X	X	X		269.4

Supplementary Table S5. Distance models for each bee species for year 2, with AIC and BIC values. An X indicates that the variable is included in the model. The variables patch and foraging bout are random variables. The variable clip is added either as a random variable, where in combination with random foraging bout, it creates a model that is sometimes called the “compound symmetry” model or as an AR(1) repeated measure structure, described in the text. Significant variables are followed by an asterisk with *: 0.1 ≤ p <0.05; **: 0.001 ≤ p < 0.01.

Flower	Patch	Foraging bout	AR (1)	Clip	AIC
		Bumble bee			
X**		X*			2014.6
X**	X	X*			2014.6
X**		X*	X		2015.7
X**	X	X*	X		2015.7
X**	X	X*		X	2015.8
X**		X*		X	2015.8
X**	X		X**		2021.4
X**			X**		2021.4
X**	X				2026.2
X**	X			X	2027.5
X**				X	2027.5
		Honey bee			
X*			X*		1028.2
X*		X	X*		1028.2

X*	X	X	X*		1028.2
X*	X		X**		1030
X*	X	X		X	1032.5
X*	X	X			1032.5
X*		X			1032.5
X*		X		X	1032.5
X*				X	1035.8
X*	X				1036.8
X*	X			X	1036.8
Leafcutting bee					
X	X	X		X	519.9
X	X			X	519.9
X	X				519.9
X				X	519.9
X	X	X			519.9
X		X			519.9
X		X		X	519.9
X	X		X		521.4
X	X	X	X		521.4
X		X	X		521.4
X			X		521.4

Supplementary Table S6. Testing the four models of bee movement for bumble bees and honeybees when the sample size for bumble bees is the same as for honey bees. The one tailed probability values (p) are presented for the randomization tests. A low probability value indicates that the model is not a good fit to the data. For Random Distance or Random Direction, a distance or direction was randomly selected from the empirical distribution of distances or directions. Modeled Distance used the best model to describe distance and Modeled Direction used the transition vector (see Table 1 and text for details). The different seeds represent different starting points and test the robustness of the different models.

		Year 1		Year 2	
Model	Seed	Bumble bee	Honey bee	Bumble bee	Honey bee
RDistance RDirection	1	0.116	0.440	0.120	0.446
	2	0.106	0.432	0.102	0.460
	3	0.09	0.434	0.138	0.442
	4	0.094	0.420	0.110	0.492
	5	0.084	0.428	0.130	0.500
RDistance MDirection	1	0.266	0.468	0.176	0.470
	2	0.312	0.416	0.204	0.484
	3	0.262	0.462	0.240	0.474
	4	0.282	0.452	0.220	0.452

	5	0.224	0.438	0.222	0.426
MDistance RDirection	1	0.424	0.378	0.494	0.352
	2	0.440	0.380	0.482	0.374
	3	0.428	0.382	0.496	0.344
	4	0.478	0.328	0.466	0.370
	5	0.434	0.370	0.492	0.388
MDistance M Direction	1	0.430	0.368	0.382	0.276
	2	0.360	0.382	0.400	0.314
	3	0.396	0.356	0.360	0.282
	4	0.378	0.330	0.428	0.288
	5	0.394	0.368	0.400	0.314
